# Extracellular Pneumococcal Serine Proteases Affect Nasopharyngeal Colonization

**DOI:** 10.3389/fcimb.2020.613467

**Published:** 2021-02-15

**Authors:** Murtadha Q. Ali, Thomas P. Kohler, Gerhard Burchhardt, Andreas Wüst, Nadin Henck, Robert Bolsmann, Franziska Voß, Sven Hammerschmidt

**Affiliations:** Department of Molecular Genetics and Infection Biology, Interfaculty Institute of Genetics and Functional Genomics, Center for Functional Genomics of Microbes, University of Greifswald, Greifswald, Germany

**Keywords:** pneumococci, serine proteases, colonization, adherence, pneumonia

## Abstract

*Streptococcus pneumoniae* has evolved versatile strategies to colonize the nasopharynx of humans. Colonization is facilitated by direct interactions with host cell receptors or *via* binding to components of the extracellular matrix. In addition, pneumococci hijack host-derived extracellular proteases such as the serine protease plasmin(ogen) for ECM and mucus degradation as well as colonization. *S. pneumoniae* expresses strain-dependent up to four serine proteases. In this study, we assessed the role of secreted or cell-bound serine proteases HtrA, PrtA, SFP, and CbpG, in adherence assays and in a mouse colonization model. We hypothesized that the redundancy of serine proteases compensates for the deficiency of a single enzyme. Therefore, double and triple mutants were generated in serotype 19F strain EF3030 and serotype 4 strain TIGR4. Strain EF3030 produces only three serine proteases and lacks the SFP encoding gene. In adherence studies using Detroit-562 epithelial cells, we demonstrated that both TIGR4Δ*cps* and 19F mutants without serine proteases or expressing only CbpG, HtrA, or PrtA have a reduced ability to adhere to Detroit-562 cells. Consistent with these results, we show that the mutants of strain 19F, which preferentially colonizes mice, abrogate nasopharyngeal colonization in CD-1 mice after intranasal infection. The bacterial load in the nasopharynx was monitored for 14 days. Importantly, mutants showed significantly lower bacterial numbers in the nasopharynx two days after infection. Similarly, we detected a significantly reduced pneumococcal colonization on days 3, 7, and 14 post-inoculations. To assess the impact of pneumococcal serine proteases on acute infection, we infected mice intranasally with bioluminescent and invasive TIGR4 or isogenic triple mutants expressing only CbpG, HtrA, PrtA, or SFP. We imaged the acute lung infection in real-time and determined the survival of the mice. The TIGR4*lux* mutant expressing only PrtA showed a significant attenuation and was less virulent in the acute pneumonia model. In conclusion, our results showed that pneumococcal serine proteases contributed significantly to pneumococcal colonization but played only a minor role in pneumonia and invasive diseases. Because colonization is a prerequisite for invasive diseases and transmission, these enzymes could be promising candidates for the development of antimicrobials to reduce pneumococcal transmission.

## Introduction

*Streptococcus pneumoniae* (pneumococcus) is a Gram-positive, facultative human pathogen, and colonizes asymptomatically and highly successful mucosal epithelial surfaces of the upper respiratory tract (URT) (Kadioglu et al., 2008; Hilleringmann et al., 2015). However, under certain conditions, when the immune system is compromised, pneumococci can disseminate from the nasopharynx into the lung and blood to cause invasive diseases, including pneumonia, meningitis, and sepsis (Song et al., 2013; WHO, 2019). Despite the development of antimicrobial therapies, vaccines, and the use of antibiotics, pneumococcal diseases remain a major threat to humans (WHO, 2019). The burden of the disease continues to be high in both industrialized and developing countries. In 2015, approximately 300,000 children under the age of 5 years died from pneumococcal related disease globally (Wahl et al., 2018). Importantly, pneumococci have to avoid entrapment in the mucus and clearance by the host immune system (Bergmann and Hammerschmidt, 2007; Weiser et al., 2018). Consequently, pneumococci use various strategies to interact with epithelial cell surface receptors. Firstly, bacterial adhesins such as the pneumococcal surface protein C (PspC, also known as CbpA), PavB, PsrP, or other adhesive pneumococcal surface components interact directly with host cell receptors (Pracht et al., 2005; Hammerschmidt, 2006; Orihuela et al., 2009; Kanwal et al., 2017; Weiser et al., 2018). Secondly, binding to host cells is promoted by the interaction between bacterial proteins referred to as microbial surface components recognizing adhesive matrix molecules like enolase or the pneumococcal adherence and virulence factor A and B (PavA, PavB) and extracellular matrix (ECM) components such as fibronectin, vitronectin, thrombospondin-1, and plasminogen (Holmes et al., 2001; Rennemeier et al., 2007; Bergmann et al., 2009; Voss et al., 2012; Kanwal et al., 2017). Thirdly, pneumococci exploit hosts proteolytic activity such as plasmin to degrade mucosal and ECM components, thereby facilitating the tight interaction with host cells (Bergmann and Hammerschmidt, 2007; Bergmann et al., 2013).

Despite this knowledge, the contribution of pneumococcal surface proteins to colonization and dissemination to the lower respiratory tract is still a crucial issue to understand. We, therefore, hypothesized that pneumococcal extracellular serine proteases could also be important for colonization under physiologically relevant *in vivo* conditions.

Pneumococci express different types of proteases. These include the zinc metalloprotease ZmpA (also known as IgA1 protease), which interacts with the host immune system by cleaving IgA into inactive components (Proctor and Manning, 1990), and ZmpB, which is involved in the modification of pneumococcal surface proteins (Novak et al., 2000). Additionally, serine proteases can contribute to pneumococcal virulence by cleaving host proteins, such as immunoglobulins, complement compounds, and proteins of the ECM (Mann et al., 2006; Mirza et al., 2011). Serine proteases possess proteolytic activity due to the presence of the catalytic triad Ser-His-Asp (Hedstrom, 2002; Supuran et al., 2002) and have been found in many organisms (Kochan and Dawid, 2013).

Depending on the serotype, pneumococci can produce, strain-dependent, up to four different serine proteases, namely the high-temperature requirement A (HtrA) protein, the subtilase family protein (SFP), the cell wall-associated serine proteinase A (PrtA), and the choline-binding-protein G (CbpG). A common feature of these proteins is their catalytic domain and that they are secreted or located on the bacterial cell surface. Interestingly, all serine proteases of interest for this study are highly conserved among the different pneumococcal serotypes (Bethe et al., 2001; Desa et al., 2008). Some of them have already been shown to influence pneumococcal pathogenesis (Mann et al., 2006; Mirza et al., 2011; de Stoppelaar et al., 2013).

The best studied serine protease is the HtrA, which is highly conserved in many bacteria and can switch from chaperon function to protease function at high temperatures (Seol et al., 1991; Spiess et al., 1999). The pneumococcal HtrA contains no specific anchoring motif and has multifunctional roles, including facilitation of pneumococcal growth at high temperatures, resistance to oxidative stress and the control of bacteriocin activity (Fan et al., 2010). HtrA has been shown to be immunogenic and protective in mice against invasive pneumococcal diseases (Li et al., 2016). The influence of HtrA on pneumococcal pathogenesis has been addressed in several studies. For example, HtrA is considered to be one of the most important serine proteases in *S. pneumoniae* virulence as it degrades the competent stimulating peptides (CSPs) and has, therefore, an impact on pneumococcal competence (Ibrahim et al., 2004a; Ibrahim et al., 2004b; Cassone et al., 2012). Furthermore, the deficiency of HtrA in *S. pneumoniae* D39 decreased bacterial loads and inflammation in the lung after intranasal challenge (de Stoppelaar et al., 2013).

Two other serine proteases, SFP and PrtA, belong to the subtilisin family. PrtA contains a typical sortase A recognition motif at the C-terminal end, which binds covalently bound to the bacterial peptidoglycan (Bethe et al., 2001; de Stoppelaar et al., 2013). Infection experiments using

*S. pneumoniae* D39 demonstrated that SFP had only a minor effect on pneumococcal virulence and may facilitate the growth of the bacteria even after a low dose infection in the lower respiratory tract (de Stoppelaar et al., 2013). However, this was shown in the presence of all other serine proteases produced by D39. Meanwhile, PrtA has been shown to contribute to the pathogenesis of pneumococcal infections in an intraperitoneal mice infection model and a contribution to lung damage in a high dose pneumonia model (de Stoppelaar et al., 2013). In contrast, PrtA does not contribute to bacterial outgrowth in pneumococcal pneumonia (Bethe et al., 2001; de Stoppelaar et al., 2013). Other previous studies showed that PrtA was highly immunogenic and activated IL-17A response, but failed to protect against pneumococcal pneumonia in infected mice (Hsu et al., 2018). Furthermore, PrtA plays an important role in blood invasion (Mahdi et al., 2015).

The fourth pneumococcal serine protease is CbpG, which is a member of the choline-binding protein (CBP) family (Gosink et al., 2000). So far, 13 to 17 pneumococcal proteins have been identified and they share a repetitive choline-binding module (CBM). The repetitive sequences of the CBM attach CBPs non-covalently to phosphorylcholine residues of cell wall anchored teichoic acids (WTA) and membrane-anchored lipoteichoic acids (LTA) of *S. pneumoniae* (Maestro and Sanz, 2016).

Mann and co-workers (Mann et al., 2006), have shown that CbpG exists in two variants in some strains. The secreted form is without choline domain due to a premature stop codon before the choline-binding domain. The other variant is surface-attached (full-length). In addition, it could play an important role in both mucosal colonization and sepsis (Gosink et al., 2000).

So far, the individual role or the synergistic effects of pneumococcal serine proteases on colonization and subsequent dissemination into the lung or blood have not been analyzed systematically. In this sense, we report here the impact of serine proteases on epithelial adherence and nasopharyngeal colonization of *S. pneumoniae* using mutants expressing only a single or no serine protease. To assess the impact of serine proteases, *in vitro* adhesion assays and an *in vivo* murine nasopharyngeal colonization model was applied using the non-invasive serotype 19F strain EF3030 (Junges et al., 2019). In addition, we assessed the role of serine proteases on virulence and dissemination in an acute murine pneumonia model using the invasive TIGR4 strain and isogenic triple serine protease mutants.

Importantly, our findings emphasize that the loss of serine proteases reduces the adherence to human epithelial cells and the nasopharyngeal colonization of mice. However, the loss of function of pneumococcal serine proteases has only moderate effects on pneumococcal virulence as analyzed in our acute murine pneumonia model. Therefore, these proteases are most likely required to facilitate colonization. They can be promising antimicrobial candidates to prevent colonization and transmission of pneumococci.

## Materials and Methods

### Bacterial Strains and Growth Conditions

*S. pneumoniae* strains and mutants used in this study are listed in **Table 1**. The pneumococcal isolate EF3030 serotype 19F originally obtained from otitis media (Andersson et al., 1983; Junges et al., 2019) was kindly provided by Anders P. Håkansson (Lund University, Sweden), while strain TIGR4 was described earlier (Tettelin et al., 2001). Growth of parental pneumococcal strains and isogenic mutants was monitored in a complex Todd-Hewitt medium supplemented with 0.5% yeast extract (THY) (Roth, Germany) and RPMI_modi_, a chemically defined medium described earlier (Schulz et al., 2014). To prepare liquid cultures, strains were thawed from glycerol-stocks and cultured on blood agar plates (Oxoid, Germany), incubated at 37°C under 5% CO_2_ atmosphere with the appropriate antibiotics presented in **Table 1** (50 µg/ml spectinomycin, 50 µg/ml kanamycin, 5 µg/ml erythromycin, 8 µg/ml chloramphenicol). Pneumococcal growth was monitored by measuring the optical density at 600nm (OD_600nm_).

**Table 1:** Characteristics of the pneumococcal strains used in this study.

Strain no.^1^	Genotype (gene locus tag)	Resistance^2^	Phenotype	Reference
***Streptococcus pneumoniae:***				
**EF3030**	19F_EF3030	None	*-*	(Andersson et al., 1983)
**PN762**	19F_EF3030Δ*htrA* (*EF3030_11105*)	Cm^r^	*-*	This work
**PN763**	19F_EF3030Δ*prtA* (*EF3030_03025*)	Erm^r^	*-*	This work
**PN769**	19F_EF3030Δ*cbpG* (*EF3030_01920*)	Spec^r^	*-*	This work
**PN768**	19F_EF3030Δ*cbpG* (*EF3030_01920*)	Erm^r^	*-*	This work
**PN765**	19FΔ*htrA*Δ*cbpG* (*EF3030_11105, EF3030_01920*)	Cm^r^, Spec^r^	*prtA*+	This work
**PN770**	19F_EF3030Δ*prtA*Δ*htrA (EF3030_03025, EF3030_11105*)	Erm^r^, Cm^r^	*cbpG*+	This work
**PN766**	19F_EF3030Δ*prtA*Δ*cbpG (EF3030_03025, EF3030_01920*)	Erm^r^, Spec^r^	*htrA+*	This work
**PN767**	19F_EF3030Δ*htrA*Δ*cbpG*Δ*prtA* (*EF3030_11105, EF3030_01920, EF3030_03025*)	Cm^r^, Spec^r^, Erm^r^	*All proteases*	This work
**PN259**	TIGR4Δ*cps*	Km^r^	*-*	(Saleh et al., 2014)
**PN494**	TIGR4Δ*cps*Δ*prt*A (*sp_0641*)	Erm^r^, Km^r^	*-*	This work
**PN488**	TIGR4Δ*cps*Δ*htr*A (*sp_2239*)	Erm^r^, Km^r^	*-*	This work
**PN663**	TIGR4Δ*cps*Δ*cbpG* (*sp_0390*)	Km^r^, Erm^r^	*-*	This work
**PN674**	TIGR4Δ*cps*Δ*sfp* (*sp_1954*)	Km^r^, Spec^r^	*-*	This work
**PN681**	TIGR4Δ*cps*Δ*htr*AΔ*sfp*	Km^r^, Erm^r^, Cm^r^	*prtA+ cbpG^+^*	This work
**PN530**	TIGR4Δ*cps*Δ*prt*AΔ*htr*A	Km^r^, Erm^r^, Cm^r^	*sfp+ cbpG^+^*	This work
**PN682**	TIGR4Δ*cps*Δ*prt*AΔ*sfp*	Km^r^, Erm^r^, Cm^r^	*htrA+ cbpG^+^*	This work
**PN692**	TIGR4Δ*cps*Δ*htrA*Δ*cbpG*Δ*sfp* (*sp_2239, sp_0390, sp_1954*)	Km^r^, Erm^r^, Cm^r^, Spec^r^	*prtA*+	This work
**PN685**	TIGR4Δ*cps*Δ*prtA*Δ*htrA*Δ*sfp* (*spd_0558*, *sp_2239, sp_1954*)	Km^r^, Erm^r^, Cm^r^, Spec^r^	*cbpG*+	This work
**PN693**	TIGR4Δ*cps*Δ*prtA*Δ*cbpG*Δ*sfp* (*spd_0558, sp_0390, sp_1954*)	Km^r^, Erm^r^, Cm^r^, Spec^r^	*htrA*+	This work
**PN695**	TIGR4Δ*cps*Δ*htr*AΔ*prt*AΔ*cbp*G (*sp_2239, spd_0558, sp_0390*)	Km^r^, Erm^r^, Cm^r^, Spec^r^	*sfp*+	This work
**PN315**	TIGR4*lux*	Km^r^	–	(Saleh et al., 2014)
**PN675**	TIGR4*lux*Δ*sfp* (*sp_1954*)	Km^r^, Spec^r^	–	This work
**PN665**	TIGR4*lux*Δ*cbpG* (*sp_0390*)	Km^r^, Erm^r^	–	This work
**PN489**	TIGR4*lux*Δ*htr*A (*sp_2239*)	Erm^r^, Km^r^	–	This work
**PN495**	TIGR4*lux*Δ*prt*A (*sp_0641*)	Erm^r^, Km^r^	–	This work
**PN750**	TIGR4*lux*Δ*prtA*Δ*cbpG* (*sp_0641, sp_0390*)	Km^r^, Erm^R^, Spec^r^	*htrA+* *sfp*+	This work
**PN743**	TIGR4*lux*Δ*sfp*Δ*htr*A (*sp_1954, sp_2239*)	Km^r^, Spec^r^, Cm^r^	*cbpG+ prtA+*	This work
**PN531**	TIGR4*lux*Δ*prt*AΔ*htr*A (*sp_0641*, *sp_2239*)	Km^r^, Erm^r^, Cm^r^	*sfp*+ *CbpG+*	This work
**PN747**	TIGR4*lux*Δ*htrA*Δ*cbpG*Δ*sfp* (*sp_2239, sp_0390, sp_1954*)	Km^r^, Erm^r^, Cm^r^, Spec^r^	*prtA*+	This work
**PN686**	TIGR4*lux*Δ*htrA*Δ*prtA*Δ*sfp* (*sp_2239, sp_0641, sp_1954*)	Km^r^, Erm^r^, Cm^r^, Spec^r^	*cbpG*+	This work
**PN752**	TIGR4*lux*Δ*prtA*Δ*cbpG*Δ*sfp* (*sp_0641, sp_0390, sp_1954*)	Km^r^, Erm^r^, Cm^r^, Spec^r^	*htrA*+	This work
**PN760**	TIGR4*lux*Δ*htr*AΔ*prt*AΔ*cbp*G (*sp_2239*, *sp_0641*, *sp_0390*)	Km^r^, Erm^r^, Cm^r^, Spec^r^	*sfp*+	This work

^1^Numbering is the stock list of the Department of Molecular Genetics and Infection Biology, University of Greifswald.

^2^Erm: Erythromycin, Km: Kanamycin, Cm: Chloramphenicol, Amp: Ampicillin, R: resistance.

### Construction of Pneumococcal Mutants

The plasmids and oligonucleotide primers used in this study are listed in **Tables 2** and **3**. Single, double, and triple *serine protease* gene deletion mutants were generated by insertion-deletion mutagenesis in *S. pneumoniae* 19F EF3030, and in TIGR4 strains (non-encapsulated Δ*cps*, and bioluminescent *lux* strains) (Schulz et al., 2014). Briefly, for the deletion of *cbpG* (*sp_0390* in TIGR4 and *EF3030_01920* in 19F), primer pair, P1421/1422 was designed to amplify the gene, including 500 bp up-and downstream using TIGR4 chromosomal DNA as a template. The PCR product was cloned into plasmid pSP72D cleaved with *Eco*RV, which contains a modified poly-linker region by deletion from the *Xho*I-*Sac*I site of pSP72 (Promega, Germany). The resulting plasmid containing the *cbpG* gene region was used as a template for an inverse PCR with the primer pair P1423/1424 to delete 642 bp of the *cbpG* gene. The primers incorporated a *Hin*dIII and *Bam*HI site for cloning of the *ermB* or *aad9* gene cassette (**Figure S1**) amplified with primers P99/100 for *ermB* and p177/118 for *aad9* to obtain plasmids pAW1100 (pSP72DΔ*cbpG*::Erm^r^) and pAW1101 (pSP72DΔ*cbpG*::Spec^r^). For the deletion of *sfp* in TIGR4 (*sp*_1954), primers P1284/P1285 were used to amplify the *sfp* gene region, including up-and downstream sequences from TIGR4. The PCR product was cloned into the *Eco*RV restriction site of pSP72D. The recombinant plasmid was used as a template for an inverse PCR with primer pair P1286/1287 containing *Bam*HI and *Hin*dIII restriction sites to delete the complete *sfp* gene sequence from the plasmid. After digestion with *Bam*HI and *Hin*dIII, the antibiotic resistance gene cassette *ermB*, *aad9* or *cat* were separately ligated to generate the plasmids pRB1119 (pSP72DΔ*sfp*::Erm^r^), pRB1132 (pSP72DΔ*sfp*::Spec^r^), and pRB1131 (pSP72DΔ*sfp*::Cm^r^), which were used to delete *sfp* in TIGR4. The *sfp* gene is not present in strain EF3030 (**Figure S2**). To construct a plasmid for the *htrA* gene deletion, the encoding gene sequence (*sp_2239 in* TIGR4 and *EF3030_11105* in 19F) of the *htrA* gene region was cloned into the *Eco*RV site of pSP72D after PCR amplification with upstream and downstream sequences using primer pair P1061/P1062. For the inverse PCR primers, P1063/1064 containing *Eco*RI restriction sites were used to delete parts of the *htrA* sequence (1104 bp) in the plasmid. The antibiotic *cat* gene cassette was amplified using primers P158/159, digested with *Eco*RI and cloned into the digested plasmid resulting in pNM991 (pSP72DΔ*htrA*::Cm^r^) (**Figure S3**). Due to the large size of *prtA* (*sp_0641* and *EF3030_03025* in 19F), only 500 bp upstream and downstream of the *prtA* gene were amplified with primers P1073/1074 (containing *Bam*HI/*Sac*I restriction sites) for the 5’-region and P1075/1076 (containing *Sac*I/*Sal*I restriction sites) for the 3’-region. The resulting PCR fragments were cloned into the *Bam*HI/*Sal*I digested vector pUC18. The recombinant plasmid was cleaved with *Ecl*136II for the insertion of the *ermB* antibiotic resistance gene cassette resulting in plasmid pGB1019 (pUC18Δ*prtA*::Erm^r^) (**Figure S4**). *S. pneumoniae* were transformed with the recombinant plasmids to delete the serine protease genes by homologous recombination as described (Hammerschmidt et al., 1997). The recombinant pneumococcal strains lacking one serine protease gene were selected on blood agar plates (Oxoid) with an appropriate antibiotic and confirmed by PCR. Finally, the recombinant plasmids pAW1100, pAW1101, pRB1131, pRB1132, pRB1119, pNM991, and pGB1019, were used to transform pneumococci to generate double and triple serine protease deficient mutants.

**Table 2:** Plasmids used in this study.

Plasmid	Vector name	Properties	Resistance^2^	Reference
**SP72**		Cloning vector	Ap^r^	Promega
**pSP72D**		Cloning vector for PCR products (derivative of pSP72)	Ap^r^	This work
**pGB1019**	pUC18Δ*prtA*::Erm^r^	pUC18 vector with *sp_0641* (*prtA*) gene partial deleted replaced with Erm^r^ resistance gene cassette, (4861 bp.)	Ap^r^, Erm^r^	This work
**pAW1101**	pSP72DΔ*cbpG*::Spec^r^	pSP72D vector with *sp_0390* (*cbpG*) gene partial deleted replaced with Spec^r^ resistance gene cassette, (4375 bp.)	Ap^r^, Spec^r^	This work
**pAW1100**	pSP72DΔ*cbpG*::Erm^r^	pSP72D vector with *sp_0390* (*cbpG*) gene partial deleted replaced with Erm^r^ resistance gene cassette, (4313 bp.)	Ap^r^, Erm^r^	This work
**pNM991**	pSP72DΔ*htrA*::Cm^r^	pSP72D vector with *sp_2239* (*htrA*) gene partial deleted replaced with Cm^r^ resistance gene cassette, (4923 bp.)	Ap^r^, Cm^r^	This work
**pRB1131**	pSP72DΔ*sfp*::Cm^r^	pSP72D vector with *sp_1954* (*sfp*) gene partial deleted replaced with Cm^r^ resistance gene cassette, (4926 bp.)	Ap^r^, Cm^r^	This work
**pRB1132**	pSP72DΔ*sfp*::Spec^r^	pSP72D vector with *sp_1954* (*sfp*) gene partial deleted replaced with Spec^r^ resistance gene cassette, (5029 bp.)	Ap^r^, Spec^r^	This work
**pRB1119**	pSP72DΔ*sfp*::Erm^r^	pSP72D vector with *sp_1954* (*sfp*) gene partial deleted replaced with Spec^r^ resistance gene cassette, (4968 bp.)	Ap^r^, Erm^r^	This work

**Table 3:** Primer used in this study.

Primer intended use	Primer/restriction enzyme	Sequence (5’-3’)^3^
**Primers used for the amplification of antibiotic resistance genes:**		
erythromycin (*ermB*)	Erm^r^ (*ermB*)_99 (*EcoRI*)	5’-CCCGGGGAAATTTTGATATCGATGGATCCGAATTCGACG GTTCGTGTTCGTGCTG-3’
	Erm^r^ (*ermB*)_100 (*EcoRI*)	5’-CCCGGGGAAATTTTGATATCGATAAGCTTGAATTCCCGT AGG CGCTAGGGACCTC -3’
Spectinomycin (*aad9*)	Spec^r^ (*aad9*) _118 (*HindIII*)	5’-AAAAGCTTGCTAGCAATTAGAATGAATATTTCCC-3’
	Spec^r^ (*aad9*) _117	5’-GTACAGGATCCGAATTCATCGATTTTCGTTCGTGAATAC-3
Chloramphenicol (*cat*)	BM19 Cm^r^ (*cat*)_158 (*EcoRI*)	5’-GCGCGAATTCGAAAATTTGTTTGATTTTTAATGG -3’
	BM18 Cm^r^ (*cat*)_159 (*SacI*)	5’-ATATGAGCTCGGGTTCCGAGGCTCAACGTCAA -3’
Chloramphenicol (*cat*)	BM19 Cm^r^ (*cat*)_180 (*BamHI*)	5’-GCGCGGATCCGAAAAT TTGTTTGATTTTTAATGG-3’
	BM19 Cm^r^ (*cat*)_181 (*HindIII*)	5’-GCGCAAGCTTGGGTTCCGAGGCTCAACGTCAA-3’
**Primers used for insertion-deletion mutagenesis**		
Amplification of *cbpG* (*sp_0390)* 5´ and 3´ flanking region	cbpG_1421 (*SacI*)	5’-GCGCGAGCTCGAAGGTGG TAGATTTCTTGATTC-3’
	cbpG_1422 (*SacI*)	5’-GCGCGAGCTCGTAATACA CCATCTTGACC-3’
Inverse PCR of *cbpG* (*sp_0390)* 5´ and 3´ flanking region (pSP72D vector)	cbpG_1423 (*BamHI*)	5’-GCGCGGATCCGTGAGCCG CTGTAATTAACAC-3’
	cbpG_1424 (*HindIII*)	5’-GCGCAAGCTTGGTAAGAT GCTTACAGATTG-3’
Mutations analyse of upstream region *cbpG* (*sp_0390*)	cbpG_1467	5’-GAATGGCTGAACTTAGTAT C-3’
Amplification of *sfp* (*sp_1954)* 5´ and 3´ flanking region	sfp_1284 (*SacI*)	5’-GCGCGAGCTCGGAGCAGTGTTACAAAATTC-3’
	sfp_1285 (*SacI*)	5’-GCGCGAGCTCGTTGTGGTAACCTGTTTGC-3’
Inverse PCR of *sfp* (*sp_1954)* 5´ and 3´ flanking region (pSP72D vector)	sfp_1286 (*BamHI*)	5’-GCGCGGATCCCGCTAGTCTGAGTGTGAG-3’
	sfp_1287 (*HindIII*)	5’-GCGCAAGCTTGATCAGCCCTATAATTATATG-3’
Mutations analyse of upstream region *sfp* (*sp_1954*)	sfp_1416	5’-GAACCTAATATTGGTTCAATAG-3’
Amplification of *htrA* (*sp_2239*) 5´ and 3´ flanking region	htrA_1061 (*BamHI*)	5’-CGCGCGGATCCAGTCAATTTTCTATTTATG-3’
	htrA_1062 (*PstI*)	5’-CTCACTGCAGAAGAGCTTCTAATTTCC-3’
Inverse PCR of *htrA* (*sp_2239*) *5*´ and 3´ flanking region (pSP72D vector)	htrA_1063 (*EcoRI*)	5’-CATGCGGAATTCGCTAATGACGATAACGAC-3’
	htrA_1064 (*EcoRI*)	5’-GCGCGAATTCCTTAACAAGAGTTCAGGTG-3’
Mutations analyse of upstream and downstream region *htrA* (*sp_2239*)	htrA_1088	5’-CCAGCTTTGCTATTATATTG-3’
	htrA_1089	5’-ACAGCCTTATTTCAGGCTG-3’
Amplification of *prtA* (*sp_0641*) 5´ flanking region	prtA_1073 (*BamHI*)	5’-GCACGGATCCTTAAGCCTTACTCTTAGCG-3’
	prtA_1074 (*SacI*)	GCGCGAGCTCATAAACTTTAACTTTGCTAGC
Amplification of *prtA* (*sp_0641*) 3´ flanking region	prtA_1075 (*SacI*)	GCGAGAGCTCGTTTATGTACTGAGATTAGATAG-3’
	prtA_1076 (*SaII*)	5’-GCGAGTCGACCACTTTCAGAATAAGGAGCCTG

^3^The primers were synthesized by Eurofins MWG Operon, Germany. The restriction site used for cloning are underlined.

### Pneumococcal Adherence Assays and Immunofluorescence Microscopy

Pneumococcal adherence to human nasopharyngeal epithelial Detroit-562 cells (ATCC CCL-138) was conducted as described (Bergmann et al., 2009). Briefly, epithelial cells were seeded (2×10^5^ cells per well) in 24-well tissue culture plates (Greiner Bio-One, Germany) in RPMI-1640 (HyClone™, Germany) supplemented with 10% (v/v) heat-inactivated fetal bovine serum (FBS) (Gibco, Germany), 2 mM glutamine, 1 mM sodium pyruvate, 1% HEPES (Sigma, Germany) and incubated for 24 h at 37°C and 5% CO_2_. The confluent monolayer (75–80% confluency) was washed three times with infection medium (cell culture medium containing 1% heat-inactivated FBS) and infected with the indicated 19F EF3030 and TIGR4Δ*cps* wild-type or mutant pneumococci using a multiplicity of infection (MOI) of 50 pneumococci per epithelial cell. Prior to infection, pneumococci were grown in THY to mid-log phase (OD_600_ of 0.35–0.4) and, after centrifugation, resuspended in phosphate-buffered saline (PBS, pH 7.4) and infection medium (RPMI-1640, 1% heat-inactivated FBS) at a ratio of 1:10. The infection was carried out for the indicated time points at 37°C, and 5% CO_2_ and non-adherent pneumococci were removed in three washing steps with RPMI-1640. Pneumococcal adherence was quantified by plating the attached and internalized (less than 0.1% of host cell-associated bacteria) pneumococci (Bergmann et al., 2009) on blood agar plates. The bacteria were counted using a colony counter (Bern University of Applied Sciences). Immunofluorescence microscopy was performed to visualize pneumococcal adherence to host cells. Detroit-562 cells were seeded on glass coverslips (diameter 12 mm) in wells of 24-well tissue culture plates and infected with pneumococci as described above. Staining host cell-attached pneumococci were performed as described (Jensch et al., 2010; Hess et al., 2017). In brief, infected host cells were treated after three washing steps with infection medium and fixed overnight at 4°C with 4% paraformaldehyde in PBS. Infected host cells were incubated with PBS/10% FBS for 3 h at room temperature to block unspecific antibody binding. Host cell-bound pneumococci were stained using a polyclonal anti-pneumococci IgG (1:1000) followed by anti-mouse Alexa-Fluor^®^ 488-coupled secondary antibody (green) (abcam, Germany). The actin cytoskeleton was stained with Phalloidin-iFlour^®^-594 conjugate (red) (abcam, Germany). Image acquisition was performed with a fluorescence microscope (Zeiss Axio-Observer.Z), imaging software (Zen 2.6, Zeiss, Germany). Each bar in the images represents 20 mM. All experiments were performed with three replicate wells tested for each experimental setup.

### Mouse Model of Colonization and Pneumonia

The influence of serine proteases on nasopharyngeal colonization and pneumonia was analyzed *in vivo* by applying two different mouse infection models, namely the nasopharyngeal colonization model and the acute pneumonia model. For the mouse colonization model, strain 19F_EF3030 was used, which colonizes the nasopharynx of mice while being mostly noninvasive (Briles et al., 1992; Junges et al., 2019). TIGR4 is a clinical isolate and causes severe pneumonia and invasive diseases in mice (van Ginkel et al., 2003). Female CD-1 outbred mice (age, 8–10 weeks) were purchased from Charles River, Sulzfeld, Germany. Mice were anesthesized intraperitoneally with ketamine (Ketanest S; Pfizer Pharma, Karlsruhe, Germany) and xylazine (Rompun^®^; Provet AG, Lyssach, Germany). Afterward, mice were intranasally challenged with 20 µl PBS/1% FBS containing 1 × 10^7^ bacteria of 19F_EF3030 (wild-type) or isogenic *serine protease* mutants in the colonization model. Nasopharyngeal washes (NP) and bronchoalveolar lavages (BAL) were collected at time points 2, 3, 7, and 14 days post-infection and CFU determined by plating as described previously (Cohen et al., 2012; Schulz et al., 2014). The acute pneumonia model was conducted by infecting mice intranasally with 20 µl PBS/1%FBS containing 9 × 10^7^ bacteria of bioluminescent TIGR4*lux* or isogenic triple *serine protease* mutants as described (Saleh et al., 2014). Infected mice were imaged and monitored using the IVIS^®^ Spectrum Imaging System (Caliper Life Sciences) to determine the dissemination of the bioluminescent pneumococci in the mice as described (Saleh et al., 2013; Saleh et al., 2014). Besides, the bioluminescent intensity was quantified as the total photon emission using LivingImage^®^ 4.1 software package (Caliper Life Sciences). The CFU of the infection dose was confirmed by plating serial dilutions on blood agar plates.

### Ethics Statement

All animal experiments were conducted in strict accordance with the recommendations in the Guide for the Care and Use of Laboratory Animals (National Research Council, USA), the guidelines of the ethics committee at The University of Greifswald and the German regulations of the Society for Laboratory Animal Science (GVSOLAS) and the European Health Law of the Federation of Laboratory Animal Science Associations (FELASA). All experiments were approved by the Landesamt für Landwirtschaft, Lebensmittelsicherheit und Fischerei Mecklenburg–Vorpommern (LALLFV M-V, Rostock, Germany) and the LALLFV M-V ethical board (LALLF M-V permit no. 7221.3-1-056/16). All efforts were made to minimize suffering, ensure the highest ethical standard and adhere to the 3R principle (reduction, refinement and replacement).

### Statistical Analysis

Statistical significance between different groups was calculated using a one-way ANOVA (Kruskal-wallis test) followed by Dunnett’s post-test for the mouse colonization model and bioluminescence measurements in the acute pneumonia model. Unpaired two-tailed Students t-test (Mann-Whitney test) was performed to analyze the difference between two groups. Two-way ANOVA analysis was used with the optical density for growth behavior. Kaplan-Meier survival curves of mice were compared by the log-rank (Mantel-Cox) test. A p-value of <0.05 was considered statically significant. All statistical analyses were performed using GraphPad Prism version 5.0 (GraphPad, Software, La Jolla, CA, USA).

## Results

### Bioinformatics Analysis and Genomic Organization of Serine Protease Genes

**Figure 1:**
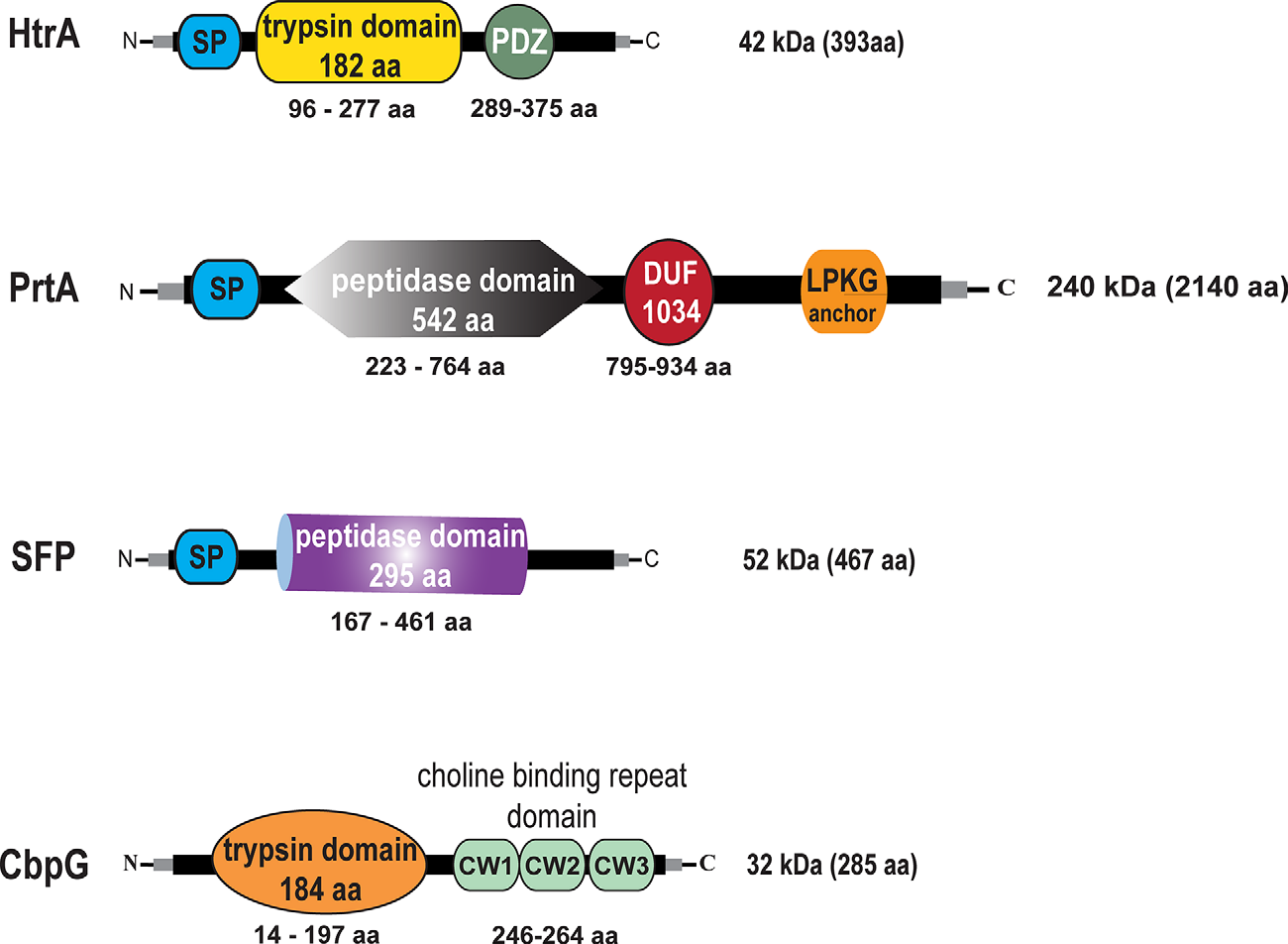
Schematic models and *in silico* analysis of pneumococcal serine proteases. High-temperature requirement A belongs to the family of trypsin-like proteases (enzymatic domain shown in yellow). PrtA (pneumococcal protease A) is a cell wall-associated serine protease (enzymatic domain in gray), which belongs to the subtilisin-like proteases with an N-terminal signal peptide (SP) and a C-terminal LPKTG sortase A anchoring motif. Subtilase family protein with the peptidase domain (purple) but lacking an anchoring motif. Choline binding protein G (enzymatic domain orange), which contains in the C-terminal part a short choline-binding module consisting of three CW-repeats.

We first analyzed pneumococcal serine protease encoding genes *in silico*. Gene and amino acid sequences were extracted from the NCBI online tool database for homolog analysis search by Clustal Omega (source data are available in supporting information). The genomic sequence of TIGR4 (ATCC BAA-334) was used as a reference sequence (Tettelin et al., 2001). Our bioinformatic analyses has been performed to provide insight into the genome organization of genes encoding serine proteases. The best known pneumococcal protein with serine proteases activity is the chaperon HtrA encoded by *sp_2239* in TIGR4 and *EF3030_11105* in 19F (protein accession numbers AAK76286.1 and QBF69928.1). HtrA consists of 393 amino acids (aa) that form a molecular weight of 42 kDa without a specific anchoring motif exhibiting two domains, the serine protease catalytic domain (residues 96–277) and the C-terminal PDZ domain (residues 289–375) (postsynaptic density protein, Drosophila disc large tumor suppressor, and zonula occludens 1 protein) (de Stoppelaar et al., 2013). The modular organization of the HtrA protein is shown in **Figure 1**. The genomic organization of the *htrA* gene region is shown in **Figure S3A**. PrtA *sp_0641* (AAK74791.1) in TIGR4 and *EF3030_03025* (QBF68585.1) in 19F is a cell wall-anchored serine protease A belonging to the subtilisin-like proteases and consists of 2140 aa, thus having a molecular weight of 240 kDa. The genomic region of the *prtA* gene (*sp_0641*, 6423 nt) is shown in **Figure S4A**. PrtA exhibits an N-terminal signal peptide and a C-terminal LPKTG anchoring motif. The peptidase domain spanning the aa residues 223–764 contains the catalytic triad, and a DUF (the domain of the unknown function 1034) of 140 aa is localized between residues 795–934. The protein model of PrtA is illustrated in **Figure 1**. Another serine protease encoding gene in pneumococci is *sfp* (*sp_1954* in TIGR4 (ABC75782.1), which encodes a subtilase family protein. The TIGR4 *sfp* gene encodes a protein of 467 aa containing a hydrophobic N-terminal signal peptide sequence followed by the catalytic domain spanning aa 167–461 (**Figure 1**), with a molecular weight of 52 kDa and without a sortase A anchoring motif. *In silico* comparative analyses of the *sfp* gene in TIGR4 *sp_1954 sfp* (467 aa) with the complete genome of 19F_EF3030 (CP035897.1 - NCBI) strains were performed using the Clustal Omega database to check whether the *sfp* gene is located in a different locus in 19F. Remarkable, *in silico*, confirmed that the *sfp* gene (subtilase family protein) and six upstream and three downstream genes are not present in the genome of the 19F strain EF3030 (**Figure S2A**). Therefore, we could generate a full *serine proteases* deficient mutant in 19F EF3030 by deleting all the other three serine proteases (CbpG, HtrA, PrtA) genes. CbpG *sp_0390* (AAK74556.1) in TIGR4 and *EF3030_01920* (QBF69943.1) in 19F, a protein composed of 285 aa, belongs to the class of pneumococcal choline-binding proteins that are non-covalently associated with the phosphorylcholine residues of teichoic acids *via* their choline-binding module (CBM). In CbpG, the CBM consists only of three choline-binding repeats (CBR) (Maestro and Sanz, 2016). In serotype 19F strain EF3030, the CBM also consists of only 3 repeats; thus, CbpG is most likely not attached to the cell surface, because it is hypothesized that at least 4 CBRs are needed for proper attachment (Yother and White, 1994). In addition to the CBM, CbpG has a functional domain spanning amino acid residues 14–197, encoding for a trypsin type domain. The *in silico* analysis by signalP software tool (Nielsen, 2017) showed that CbpG had no recognizable signal peptide for protein secretion (**Figure 1**). The *sp_0389* gene located upstream of the *cbpG* locus encodes a hypothetical protein. In contrast, the downstream located gene *sp_0391* encodes the choline-binding protein F (Molina et al., 2009) (**Figure S1A**).

### Impact of Pneumococcal Serine Proteases on Bacterial Fitness and Growth Behavior

**Figure 2:**
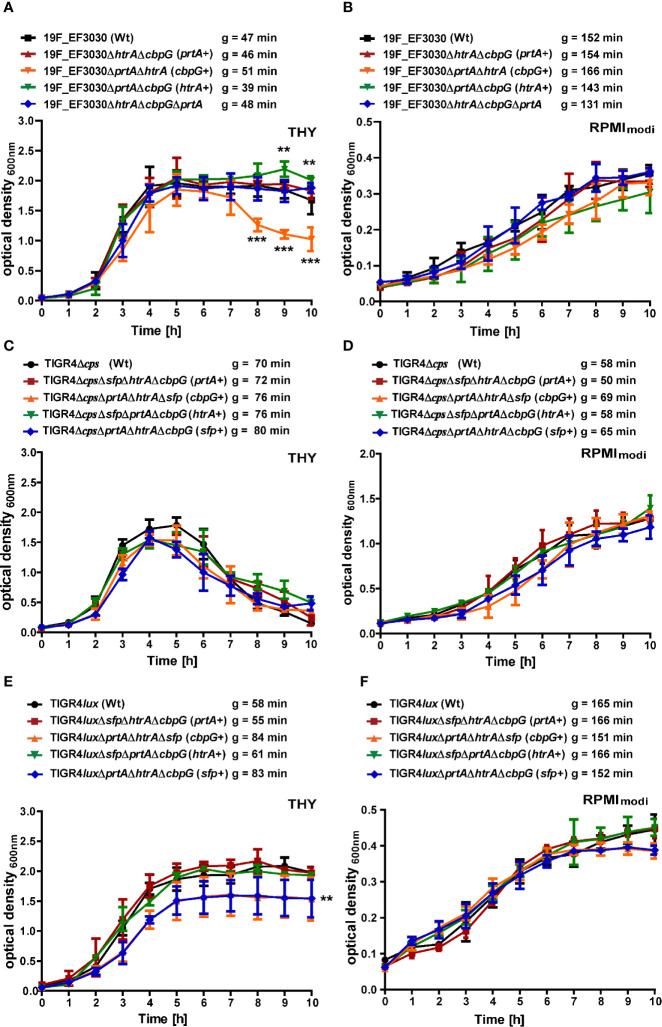
Growth behavior of serine protease deficient pneumococci. Wild-type and isogenic mutants were cultured at 37°C and 5% CO_2_ in THY and chemically defined medium (RPMI_modi_). Pneumococcal growth was monitored at OD_600_. The mean of four individual growth experiments is shown for 19F_EF3030 **(A, B)**, TIGR4Δ*cps* **(C, D)**, and TIGR4*lux* **(E, F)**. Error bars represent SD (n = 4). The symbol “g” indicates the generation time, calculated from four biological replicates. The data were statistically analyzed using a two-way ANOVA analysis *P < 0.05.

To evaluate the effect of gene knockouts of different serine proteases on pneumococcal fitness and growth, we have investigated *S. pneumoniae* serotype 19F strain EF3030 and serotype 4 strain TIGR4 and their isogenic mutant strains under two different culture conditions. Pneumococcal growth was monitored in chemically defined RPMI_modi_ or complex THY medium. Furthermore, the generation times of the wild-type and mutants were calculated in all the growth curves. In THY, the 19F wild-type strain and the *serine protease* mutants showed a comparable growth pattern, except for the mutant with CbpG+ as the only functional serine protease, which started to lyse shortly after reaching the stationary phase (**Figure 2A**). Statistically significant differences were only monitored in the late stationary phase for the CbpG+ mutants at time points 8, 9, and 10 h compared to the wild-type. In RPMI_modi,_ the 19F wild-type strain and the mutant expressing only PrtA+ had a similar growth behavior. However, 19F mutants with only one functional serine protease (CbpG+ or HtrA+) showed a delayed lag phase. Nevertheless, they reached a similar optical density compared to the wild-type (**Figure 2B**). No significant differences in the growth of the wild-type and isogenic mutants were observed in RPMI_modi_. Compared to 19F, the TIGR4Δ*cps* strain and its isogenic serine protease mutants showed similar growth behavior in complex and chemically-defined medium. However, TIGR4 and the corresponding mutants started to lyse immediately after reaching the stationary growth phase in the THY medium (**Figures 2C, D**).

However, the encapsulated TIGR4*lux* and its isogenic *serine protease* mutants exhibited an extended stationary growth in THY medium. The mutants expressing CbpG+ or SFP+ showed similar generation times compared to the parental strain, but the overall growth was significantly reduced. The mutants entered the stationary phase already at a lower optical density (**Figure 2E**). TIGR4*lux* mutants cultured in RPMI_modi_ medium showed no significant differences compared to the parental strain (**Figure 2F**).

We have further investigated growth of 19F single isogenic mutants in TYH and RPMI_modi_ medium. No growth differences were observed when compared to the wild-type 19F (**Figure S5**). To determine whether the deletion of serine proteases impacts nutrient acquisition strain 19F and TIGR4 and their the isogenic mutants expressing no serine protease (19F) or only SFP were grown in CDM (Mickelson, 1964; Leonard et al., 1970) supplemented with 2% casein hydrolysate (Härtel et al., 2011). No significant growth differences were observed between the mutants and the corresponding parental strains (**Figure S5**). Growth rates are listed in **Table S.1**.

### *Serine Proteases* Deficiency Reduced *S. pneumoniae* 19F and TIGR4Δ*cps* Adherence to Nasopharyngeal Host Cells

**Figure 3:**
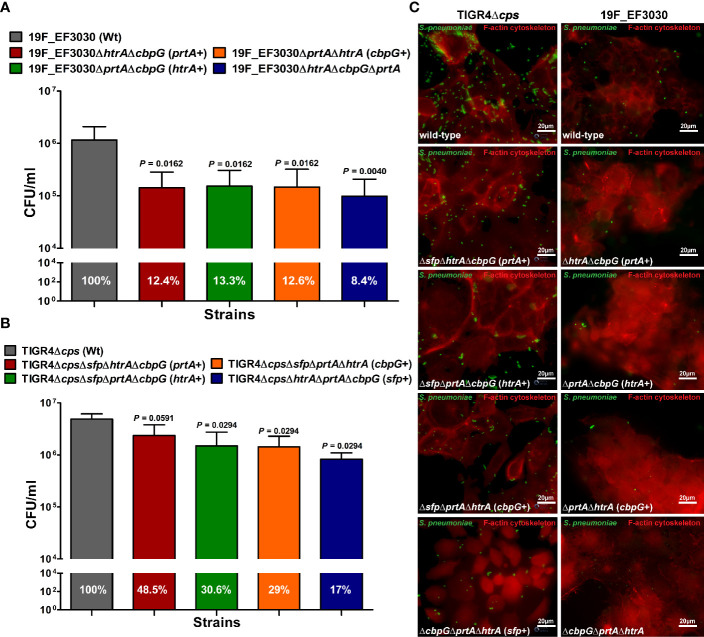
Impact of pneumococcal serine proteases on pneumococcal adherence to host epithelial cells. Human Detroit-562 cells were infected for 4 h with an MOI of 50 of *S. pneumoniae* EF3030, TIGR4Δ*cps* wild-type or mutant strain. **(A, B)** Colony-forming units were determined post-infection by plating host cell adherent pneumococci on blood agar plates. Results were presented as the mean ± SD for at least four independent experiments performed in triplicates. *n.s*. *P < 0.05, and **P ≤ 0.01 relative to the parental 19F pneumococcal strain. **(C)** Immunofluorescence microscopy of pneumococci attached to Detroit-562 cells after 4 h infection. Adherent pneumococci were stained with anti-pneumococcal antiserum followed by secondary Alexa-488 conjugated anti-IgG antibody (green). The epithelial F-actin was stained with Phalloidin-iFlour-594 conjugate (red).

The initial step of pneumococcal infection is the specific adherence to host epithelial cells of the upper respiratory tract leading to colonization (Weiser et al., 2018). We investigated extracellular serine proteases role in adherence to host epithelial cells using the human nasopharyngeal epithelial cell line Detroit-562. Capsule expression is known to have a negative effect on adherence (Kim and Weiser, 1998; Hammerschmidt et al., 2005), and the non-encapsulated TIGR4Δ*cps* shows significantly higher *in vitro* adherence to epithelial cells than the encapsulated TIGR4 strain (Bootsma et al., 2007). Therefore, the impact of serine proteases on pneumococcal adherence was studied by infecting Detroit-562 with TIGR4Δ*cps* (serotype 4) and 19F EF3030. Detroit-562 cells were infected with wild-type or isogenic *serine protease* mutants with an MOI 50 for 4 h. For 19F, double *serine protease* mutants expressing only one functional serine protease (CbpG+, PrtA+ or SFP+) or a mutant lacking all serine proteases revealed a significant reduction of 19F adherence to Detroit-562 cells in comparison to the parental strain (*P <* 0.05, and *P <* 0.01) (**Figures 3A, C**). The TIGR4Δ*cps* triple serine protease mutants with only a single functional protease showed a significant reduction of adherence compared to the parental strain (*P <* 0.05), with the exception of the PrtA positive triple knockout (**Figures 3B, C**). Thus, the results suggest a substantial role of extracellular serine proteases in adherence to epithelial cells. Therefore, our adherence data suggest that serine proteases contribute to interacting with the host respiratory epithelial cells.

### Extracellular Serine Proteases Are Involved in Pneumococcal Colonization

**Figure 4:**
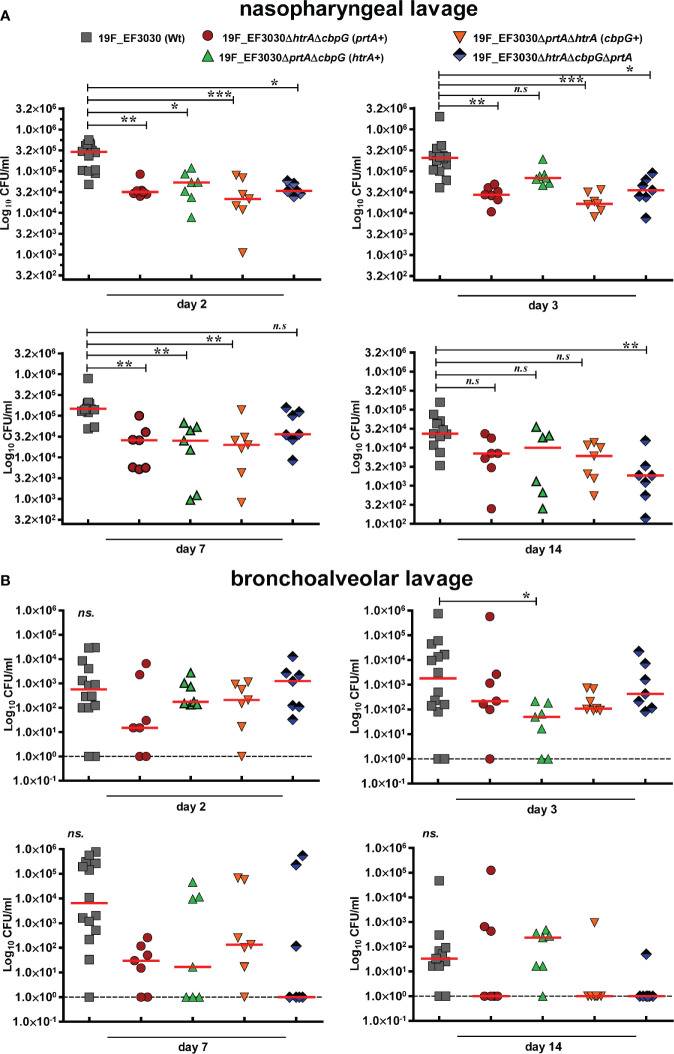
Nasopharyngeal colonization in a murine infection model. Eight to ten-week-old female CD-1 outbred mice (n = 7) were infected intranasally with a CFU of 1 × 10^7^ pneumococci of serotype 19F (EF3030) or isogenic *serine protease* mutants: 19F_EF3030Δ*htrA*Δ*cbpG* (*prtA*+), 19F_EF3030Δ*prtA*Δ*cbpG* (*htrA*+), 19F_EF3030Δ*htrA*Δ*prtA* (*cbpG*+), or 19F_EF3030Δ*htrA*Δ*prtA*Δ*cbpG*. Mice were sacrificed at day 2, 3, 7, or 14 post-infections, and pneumococci recovered by a nasopharyngeal **(A)** or bronchoalveolar lavage **(B)** and plated on blood agar plates for quantification. Results are shown as scatter plots, where each dot represents one individual mouse. The data were statistically analyzed using a Kruskal-Wallis test *p < 0.05; **p < 0.01 and ***p < 0.001. The dashed line represents the limit of detection.

We showed that loss of functional serine proteases leads to reduced adherence to human nasopharyngeal epithelial cells. We assumed therefore that *in vivo* nasopharyngeal colonization is reduced in the absence of serine proteases. Hence, the impact of pneumococcal serine proteases on nasopharyngeal colonization has been assessed in a murine colonization model. We intranasally infected female CD-1 mice (7 mice/group) with 10^7^ CFUs of either 19F EF3030 (wild-type) or isogenic *serine protease* mutants. Pneumococci were recovered 2, 3, 7, and 14 days post-infection from the nasopharyngeal cavity and lungs. In comparison to the isogenic wild-type, the deficiency in serine proteases resulted in a significant reduction (*P <* 0.01, and *P <* 0.001) of pneumococcal CFU in the nasopharyngeal cavity two days post-infection. Ten-fold less mutant pneumococci were determined after 2, 3, 7, and 14 days post-infection (**Figure 4A**).

Furthermore, on day three post-infection the bacterial load of 19F_EF3030Δ*prtA*Δ*cbpG* expressing only HtrA+ was significantly reduced in bronchoalveolar lavages. Although there was a trend to lower CFU in the lower respiratory tract, the other mutants did not show significant differences compared to the parental wild-type 19F (**Figure 4B**). In general, mice colonized with mutants deficient for serine proteases eliminated the bacteria faster on days 7 and 14 from the lower respiratory tract in comparison to the 19F wild-type. These data confirm the low invasive potential of strain 19F in the lung host compartment. Taken together, the results of the *in vitro* adherence study and the experimental mouse colonization model indicate that pneumococcal adherence to the nasopharynx is strongly affected by the loss of different serine proteases.

### An Acute Pneumonia Model Indicates Only a Moderate Effect of Serine Proteases on *S. pneumoniae* TIGR4 Virulence

We further investigated the role of serine protease deficiency on pneumococcal virulence using an acute pneumonia model in mice. Because reduced colonization also leads to lower infection severity of the lungs, we hypothesized that the development of acute lung infection will be prevented in the absence of serine proteases. We infected 8–10-week old female CD-1 outbred mice (14 mice/group) intranasally with 9 × 10^7^ bioluminescent TIGR4*lux* or corresponding isogenic triple serine protease knockout strains expressing only one out of four functional serine protease. We monitored the influence of serine proteases on pneumococcal dissemination into the lungs and transcytosis of the respiratory epithelial barrier into the bloodstream *in vivo* using the IVIS^®^ Spectrum bioimaging system. Mice infected with the parental TIGR4*lux* strain showed the first weak signs of pneumonia in the lung after 24 h.

**Figure 5:**
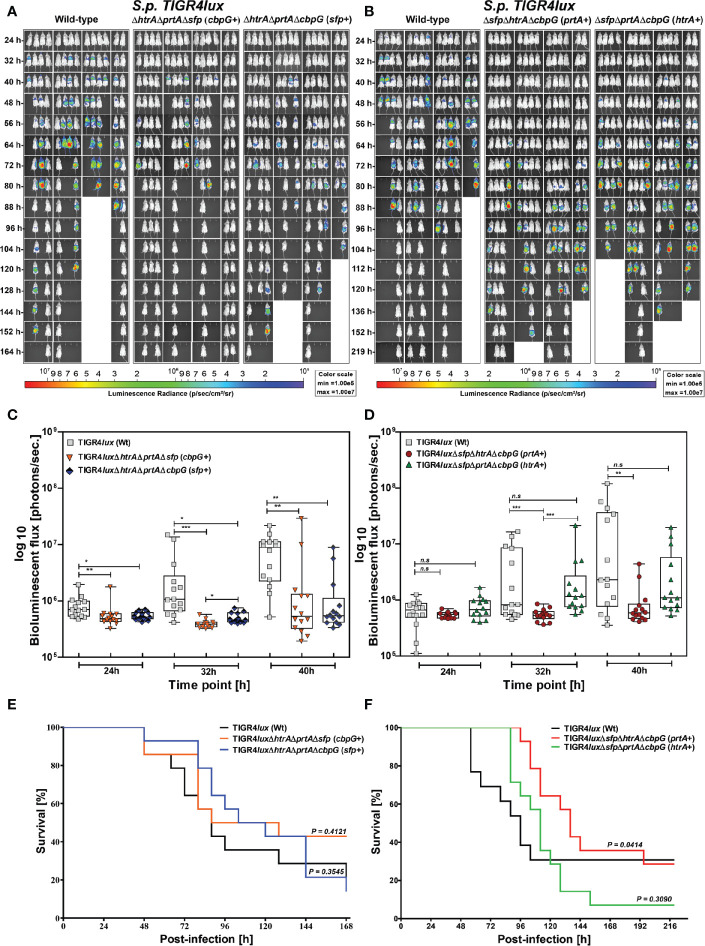
Acute infections in an experimental pneumonia model. Eight to ten-week-old female CD-1 mice (n = 14) were intranasally infected with *S. pneumoniae* TIGR4*lux* wild-type or isogenic triple *serine protease* mutants using an infection dose of 9×10^7^ CFU. **(A, B)** The course of infection was monitored in real-time using the IVIS^®^-Spectrum *in vivo* bioimaging system. **(C, D)** The multiplication and dissemination of bioluminescent pneumococci in infected mice were quantified at indicated time points by measuring the luminescence intensity (photons/second). Data are shown as a Box-Whisker graph showing the values for each mouse. Kruskal-Wallis test was used for statistical analysis. **(E, F)** Kaplan-Meier survival curves of mice infected with *S. pneumoniae* TIGR4*lux* or *serine protease* deficient mutants. The log-rank (Mantel-Cox) test was used for statistical analysis. *p < 0.05; **p < 0.01 and ***p < 0.001.

On the contrary, the lung infection of mice infected with triple mutants, lacking the expression of three out of four serine proteases, started earliest 40 h post-infection (**Figures 5A, B**). Besides, the overall bioluminescent intensity was significantly decreased for all mice infected with mutants expressing only PrtA+, CbpG+ or SFP+ (**Figures 5C, D**). However, the bioluminescent flux of mice infected with the mutant expressing HtrA+ showed no significant difference compared to the wild-type (**Figure 5D**). The survival of mice infected with triple *serine protease* mutants expressing only one functional protease (CbpG+, SFP+, or HtrA+) showed, except for the PrtA+ mutant, no differences and are comparable to the parental strain TIGR4*lux* (**Figures 5E, F**). The triple mutant TIGR4 PrtA+ was significantly attenuated (*p-value* 0.0414). For example, only one mouse out of fourteen developed severe pneumonia 64 h post-infection as visualized by bioimaging (**Figure 5B**). Indeed, our results of the real-time bioimaging with the PrtA+ expressing TIGR4 mutant showing the lowest bioluminescence correlates with the survival time of the mice (**Figures 5C, F**). Taken together, the loss of serine proteases did not dramatically affect the virulence of TIGR4*lux* in the acute mouse pneumonia model. The only exception was the PrtA positive TIGR4 triple knockout, which was significantly attenuated, pointing to an important role of PrtA in the acute pneumonia model.

## Discussion

Serine proteases secreted by pneumococci or bound to the cell surface play a pivotal role in the pathogenesis of this human pathogen. These proteases have critical pathological and physiological functions including enzyme modification and cleavage of host immune proteins, which affect colonization and evasion of the host defense (Supuran et al., 2002; Bergmann and Hammerschmidt, 2007; Janoff et al., 2014). This has been intriguingly shown for zinc-metalloproteases (Novak et al., 2000; Govindarajan et al., 2012). Other studies have also assessed the impact of serine proteases on pneumococcal virulence using different experimental infection models (**Table 4**). In these studies the pneumococcal mutants were deficient for only one of the serine proteases (Gosink et al., 2000; Bethe et al., 2001; Ibrahim et al., 2004a; Mann et al., 2006; Mirza et al., 2011; de Stoppelaar et al., 2013; Mahdi et al., 2015; Hsu et al., 2018). Hence, a potential redundancy of their mode of action could not be finally excluded. However, the lack of a functional HtrA attenuated *S. pneumoniae* D39 in the acute mouse pneumonia model and leads to lower bacterial burden in the lung, blood and organs. This indicated the crucial role for virulence, while the lack of PrtA had only a minor effect (de Stoppelaar et al., 2013). Similar, our studies with TIGR4 demonstrated that only mutants expressing HtrA showed a similar bioluminescence of the infected lungs post-intranasal infection, suggesting similar bacterial burden in the lungs (**Figures 5C, D**). Recently, the important role of the chaperone/protease HtrA in bacto-viral co-infections was shown. HtrA was highly expressed under influenza A virus induced inflammation and protects against opsonophagocytosis and mediates resistance against oxidative damage (Sender et al., 2020). Thus, HtrA of pneumococci but also of other bacterial species is an indispensable virulence factor (**Table 4**).

**Table 4:** Bacterial serine proteases and their role in pathogenicity.

Serine Proteases	Bacterial Species	Associated Disease	Role in Pathogenesis	Host-Targets	References
**PrtA**	*Streptococcus pneumoniae*	CAP^1^, sepsis, meningitis	killing by apolactoferrin colonization adherence, pneumonia	cleaves human apolactoferrin	(Bethe et al., 2001; Mirza et al., 2011), **this study**
**CbpG**	*Streptococcus pneumoniae*	CAP, sepsis, meningitis	adherence, virulence factor adherence, colonization	cleaves human fibronectin	(Gosink et al., 2000; Mann et al., 2006), **this study**
**SFP**	*Streptococcus pneumoniae*	CAP, sepsis, meningitis	facilitates bacterial growth, adherence, colonization	unknown function	(de Stoppelaar et al., 2013), this study
**CspA** **(**SFP homolog)	*Streptococcus agalactiae*	CAP, sepsis, and meningitis	virulence factor, resistance to opsonophagocytosis	cleaves human fibronectin, inactivates chemokines	(Harris et al., 2003; Bryan and Shelver, 2009)
**HtrA** **chaperone/protease**	*Streptococcus pneumoniae*	CAP, sepsis, meningitis	chaperone, heat-shock protein, protease, virulence factor, competence pathways, growth advantage in influenza A virus co-infections adherence, colonization	quality control of secreted proteins	(Ibrahim et al., 2004b; Sebert et al., 2005; Cassone et al., 2012; de Stoppelaar et al., 2013; Kochan and Dawid, 2013; Sender et al., 2020), **this study**
	*Streptococcus pyogenes*	purulent diseases of the pharynx and skin	processing of extracellular virulence factors and haemolytic activity	cleavage of complement factor C5a	(Wexler et al., 1985; Lyon and Caparon, 2004)
	*Streptococcus mutans*	dental caries	colonization	biofilm formation	(Biswas and Biswas, 2005)
	*Campylobacter jejuni*	Campylo-bacteriosis, Guillian Barré syndrome	bacterial adhesion, transmigration and invasion	cleavage of E−cadherin, apoptosis, and immune responses	(Boehm et al., 2013; Boehm et al., 2018)
	*Helicobacter pylori*	gastritis, ulcers symptoms	bacterial transmigration, activation of type IV secretion	cleavage of occludin, claudin‐8, E‐cadherin and fibronectin	(Hoy et al., 2010; Schmidt et al., 2016; Tegtmeyer et al., 2017)

^1^community-acquired pneumonia.

However, the role of pneumococcal serine proteases including HtrA on colonization is still unknown. To investigate the influence of pneumococcal serine proteases on the host-pathogen interaction in the upper respiratory tract, we have studied the effect on adherence and colonization in the genetic background of triple protease deletion mutants. For adherence and nasopharyngeal colonization studies, we have used the serotype 19F (strain EF3030) (Junges et al., 2019), while the invasive TIGR4 strain was used in the acute pneumonia model. The *S. pneumoniae* serotype 19F strain EF3030 described as the causative agent for otitis media has already been shown to be an efficient colonizer in murine model systems (Joloba et al., 2001; Blevins et al., 2014). Our results showed that adherence of *S. pneumoniae* 19F to host epithelial cells is affected in a mutant deficient for two or three serine proteases produced by wild-type 19F and TIGR4Δ*cps*. This finding is in accordance with other studies showing that already the deletion of HtrA or homologs in other species than pneumococci leads to decreased bacterial adhesion to epithelial cells (Brondsted et al., 2005; Frees et al., 2013). The underlying molecular mechanisms in pneumococci are so far not fully understood. However, HtrA homologs in other bacterial species were involved in processing of adhesins and thus in the activity of adhesins (Backert et al., 2018). Furthermore, CbpG is proposed to be a multifunctional protein cleaving ECM proteins and is involved in adherence as indicated by a reduced adherence of a TIGR4 *cbpG*-mutant to nasopharyngeal epithelial cells (Gosink et al., 2000; Mann et al., 2006). Thus, each serine protease might contribute to pneumococcal adherence and the expression of a single serine proteases is probably not sufficient to reach adherence comparable to the wild-type. However, the molecular mechanisms might differ and have to be explored in further studies. Because of the altered adherence of the mutants under *in vitro* infections, we hypothesized that the loss of serine proteases would also have an impact on pneumococcal colonization of the nasopharynx. Hence, the experimental nasopharyngeal colonization model was used to assess whether the diminished adhesion of the 19F mutants to human epithelial cells correlates with the inability of serine protease deficient mutants to colonize the murine nasopharynx. Indeed, we monitored a dramatic decrease in the bacterial loads of the serine protease mutants in the nasopharyngeal colonization model compared to the isogenic parental strain 19F. Thus, these data strongly suggest that serine proteases are indispensable for colonization. Interestingly this effect is more pronounced in the nasopharynx compared to the bronchoalveolar space, which is not surprising considering the low capacity of 19F to cause lung infections in the mouse pneumonia model (Marks et al., 2013; Junges et al., 2019). In addition, the double mutant expressing HtrA shows 14 days post-infection a similar behavior in the bronchoalveolar lavage compared to the wild-type 19F, despite early time points show a significant reduction in colonization.

When using TIGR4 in the acute pneumonia model our data show that the deficiency of three serine proteases did not impair the full virulence of TIGR4 in mice. However, the quantification of bioluminescence as well as the monitoring of mouse survival suggest that the triple knockouts have a slightly reduced capacity to cause pneumonia and, in consequence, invasive disease. The only exception is the mutant with a functional PrtA, because this mutant shows a significant attenuation in the acute pneumonia model. This is an interesting finding because the PrtA positive mutant is deficient in HtrA. The protein HtrA was shown earlier to be a major virulence factor in pneumococcal pneumonia caused by *S. pneumoniae* D39 in C57BL/6 mice, while SFP and PrtA played no major role (de Stoppelaar et al., 2013). It has to be mentioned that C57BL/6 are more resistant to pneumococcal infections compared to CD-1 mice (Gingles et al., 2001) used in our study and that, despite being able to cause severe pneumonia in mice, D39 and TIGR4 differ in their genomic content and show different regulatory processes (Saleh et al., 2014; Schulz et al., 2014). The importance of HtrA is furthermore evident in our pneumonia model because the HtrA expressing triple *serine protease* mutant shows, according to the bioluminescence data, a similar multiplication in the lung compared to the parental TIGR4 strain. Mutants lacking HtrA show significantly lower bioluminescence compared to the isogenic parental strain TIGR4. In addition, the mouse survival rates confirm the importance of HtrA for full virulence of *S. pneumoniae* TIGR4.

Therefore, the most interesting questions for further studies are the substrate-specificity of the serine proteases and how do these proteases mechanistically contribute to colonization or adhesion. The PDZ domain of HtrA is important for protein-protein interactions and is also important for the interaction with the protease domain and hence, for the proteolytic activity of HtrA (Fan et al., 2010; Fan et al., 2011). Therefore, the underlying mechanism is likely related to the HtrA enzymatic activity function, which can be activated by the interaction with host matrix components to modify host proteins during colonization. The role of CbpG for pneumococcal adherence and binding to human cells has already been shown in previous studies (Gosink et al., 2000; Mann et al., 2006), which may also apply to the other serine proteases. CbpG was shown to degrade the host protein fibronectin and casein (Mann et al., 2006).

The cleavage or degradation of host proteins is not limited to the activity of CbpG. The cell wall anchored PrtA is involved in the cleavage of host proteins like fibrinogen or collagen in order to penetrate tissues or escape from the immune system (Frolet et al., 2010). Besides, PrtA cleaves the host protein apolactoferrin to the even more bactericidal lactoferricin facilitating killing of pneumococci (Mirza et al., 2011). Therefore, it has been suggested that the presence of PrtA in the host may reduce pneumococcal load during systemic infection in the mouse model (de Stoppelaar et al., 2013) due to the bactericidal effect of apolactoferrin (Mirza et al., 2011).

Another study showed that the deficiency of PrtA in *S. pneumoniae* D39 reduced virulence in a sepsis mouse model after intraperitoneal infection (Bethe et al., 2001). Considering our colonization data with 19F and the pneumonia data with TIGR4, we hypothesize that PrtA contributes to colonization but not to lung infections. A finding that confirms earlier data proposing that some of the sortase anchored pneumococcal proteins including PrtA have adhesive functions (Frolet et al., 2010; de Stoppelaar et al., 2013). However, to decipher the individual impact of PrtA or other serine proteases on adherence and colonization or even pneumonia single knockout strains and *in trans* complemented mutants have to be tested in adherence, colonization but also in biofilm assays. Additionally, a structure-function analysis is needed, which requires the structural analysis of serine proteases. So far, the complete structure of HtrA of *Campylobacter jejuni* is solved, while for pneumococci only the PDZ domain is reported (Fan et al., 2010; Fan et al., 2011; Zarzecka et al., 2020).

SFP has previously been shown to play only a minor role in pneumococcal virulence of strain D39 (de Stoppelaar et al., 2013). CspA, a serine protease from *S. agalactiae* (group B streptococci), is highly homologous to SFP and has been shown to inactivate chemokines (Bryan and Shelver, 2009). Importantly, *in silico* results indicated that the *sfp* gene is not present in serotype 19F strain EF3030. Furthermore, SFP is shortened in TIGR4 due to a premature stop codon. Our data confirm that SFP is probably not crucial for *S. pneumoniae* virulence, while its role in colonization is still elusive.

We postulate that extracellular serine proteases influence pneumococcal adherence to mucosal cells. Colonization is therefore also affected either by their proteolytic activity or their adhesive function. On the one hand, it is likely that serine proteases contribute to host-pathogen interactions by degrading host proteins, facilitating binding to host cells or even dissemination in the host. On the other hand, it cannot be excluded that serine proteases are also involved in the cleavage and release of other pneumococcal surface proteins, which might be a strategy to evade the host immune system and facilitate adhesion to host cells by demasking important receptors. A direct adhesive activity has already been proposed for CbpG (Mann et al., 2006). This adhesion function may also be possible for the other serine proteases but must be further investigated in future studies.

In conclusion, we highlight here that the deficiency of serine proteases impairs significantly nasopharyngeal colonization. Therefore, serine proteases have the potential to facilitate pneumococcal colonization and binding to their host targets.

## Data Availability Statement

The original contributions presented in the study are included in the article/supplementary material. Further inquiries can be directed to the corresponding author.

## Ethics Statement

The animal study was reviewed and approved by Landesamt für Landwirtschaft, Lebensmittelsicherheit und Fischerei Mecklenburg–Vorpommern (LALLFV M-V, Rostock, Germany) and the LALLFV M-V ethical board (LALLF M-V permit no. 7221.3-1-056/16).

## Author Contributions

Conceived and designed the experiments: MA and SH. Experiments performed by MA, TK, FV. Mutants constructed by MA, NH, AW, RB, and GB. Editorial advice: SH, TK, and GB. Data analyzed and wrote the manuscript: MA, and revision SH. All authors contributed to the article and approved the submitted version.

## Funding

This study was supported by the German Academic Exchange Service (DAAD) as a grant scholarship and part of the PhD thesis of MA, Funding programme/-ID: Research Grants - Doctoral Programmes in Germany, 2017/18 (57299294), ST33. The work was further supported by the Bundesministerium für Bildung und Forschung (BMBF- Zwanzig20 -InfectControl 2020 – Project VacoME – FKZ 03ZZ0816A to SH) and the Federal Excellence Initiative of Mecklenburg Western Pomerania and European Social Fund (ESF) Grant KoInfekt (ESF_14-BM-A55-0001_16). The funders had no role in study design, data collection, analysis, decision to publish, or manuscript preparation. We acknowledge support for the Article Processing Charge from the DFG (German Research Foundation, 393148499) and the Open Access Publication Fund of the University of Greifswald.

## Conflict of Interest

The authors declare that the research was conducted in the absence of any commercial or financial relationships that could be construed as a potential conflict of interest.
