# Supplementary material for: Extracellular Pneumococcal Serine Proteases Affect Nasopharyngeal Colonization
Source: Front Cell Infect Microbiol. 2021 Feb 15;10:613467. doi: 10.3389/fcimb.2020.613467 (PMC7917122; doi:10.3389/fcimb.2020.613467)
Supplement: Supplementary file 1 [file DataSheet_1.pdf]

Supplementary Material

Extracellular pneumococcal serine proteases affect nasopharyngeal colonization

Murtadha Q. Ali[1], Thomas P. Kohler[1], Gerhard Burchhardt[1], Andreas Wüst[1], Nadin Henck [1], Robert Bolsmann[1], Franziska Voß[1], and Sven Hammerschmidt[1*]

[1]Department of Molecular Genetics and Infection Biology, Interfaculty Institute of Genetics and Functional Genomics, Center for Functional Genomics of Microbes, University of Greifswald, Greifswald, Germany

*** Correspondence:**
Prof. Dr. Sven Hammerschmidt
sven.hammerschmidt@uni-greifswald.de

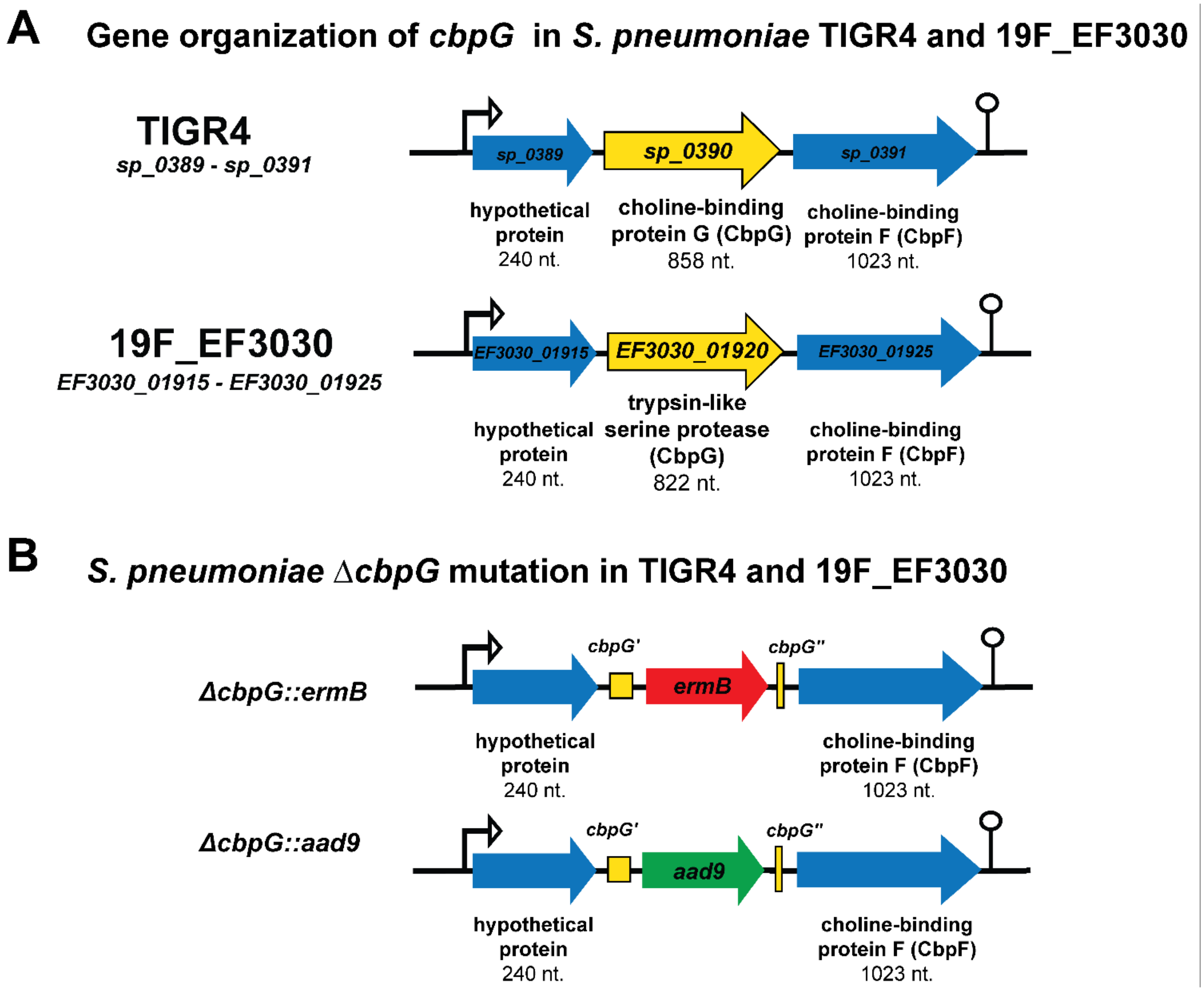

Figure S1. Genetic organization of *cbpG* in *S. pneumoniae* TIGR4 and 19F_EF3030.
(A) Chromosomal localization of the *cbpG* gene (*sp_0390*, *EF3030_01920*) shows a 99.6% homology. For the homology search, BlastN and BlastP were used (http://blast.ncbi.nlm.nih.gov/Blast.cgi). Arrowheads indicate the orientation of the genes. The Neural network predicted putative promoters (black arrowheads) by promotor prediction program. The transcription termination signals are shown as loops.
(B) Schematic model of *cbpG* mutants constructed by insertion-deletion mutagenesis shows the *erm* or *aad9* gene cassette insertion sites in the *cbpG* gene.

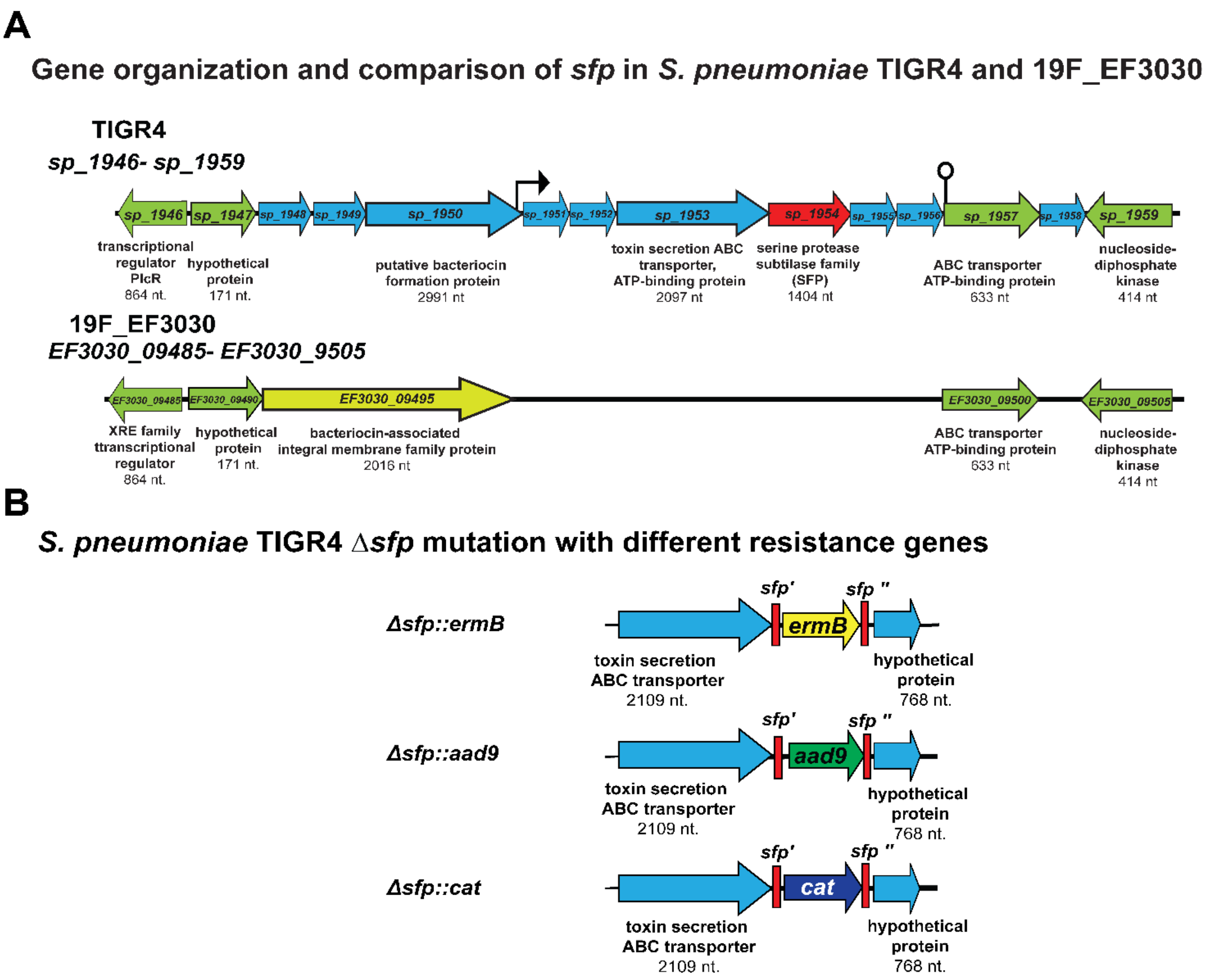

Figure S2. Genetic organization of *sfp* in *S. pneumoniae* TIGR4 and EF3030.

(A) Comparison of genome regions in TIGR4 *sp_1946-sp_1959* with 19F *EF3030_09485-EF3030_09505*. The *sfp* gene in TIGR4 *sp_1954* is shown as a red arrow with 1404 nt, while in 19F_EF3030, the *sfp* gene and 6 genes upstream are absent. The homologous analysis was done (Clustal Omega tool) to check whether the *sfp* gene is located in a different gene locus in 19F. We found that the upstream genes *sp_1947* (171 nt) and *sp_1946* (864 nt) encoding the hypothetical protein and transcriptional regulator, respectively, are homolog (100.0% identical) to the gene *EF3030_09490*, *EF3030_09485*. The genes *sp_1957* and *sp_1959* (light green) encoding for an ABC transport system (ATP-binding protein) and nucleoside-diphosphate kinase were identical to *EF3030_09500* and *EF3030_09505*, respectively. Therefore, three genes downstream of *sfp* are missing in EF3030. Arrowheads indicate the orientation of the gene, the predicted putative promoters (black arrowheads), and the transcription termination signals are shown as loops.

(B) Schematic model of *sfp* mutants constructed by insertion-deletion mutagenesis shows the *erm*, *aad9*, or *cat* genes cassette inserted in the *sfp* gene.

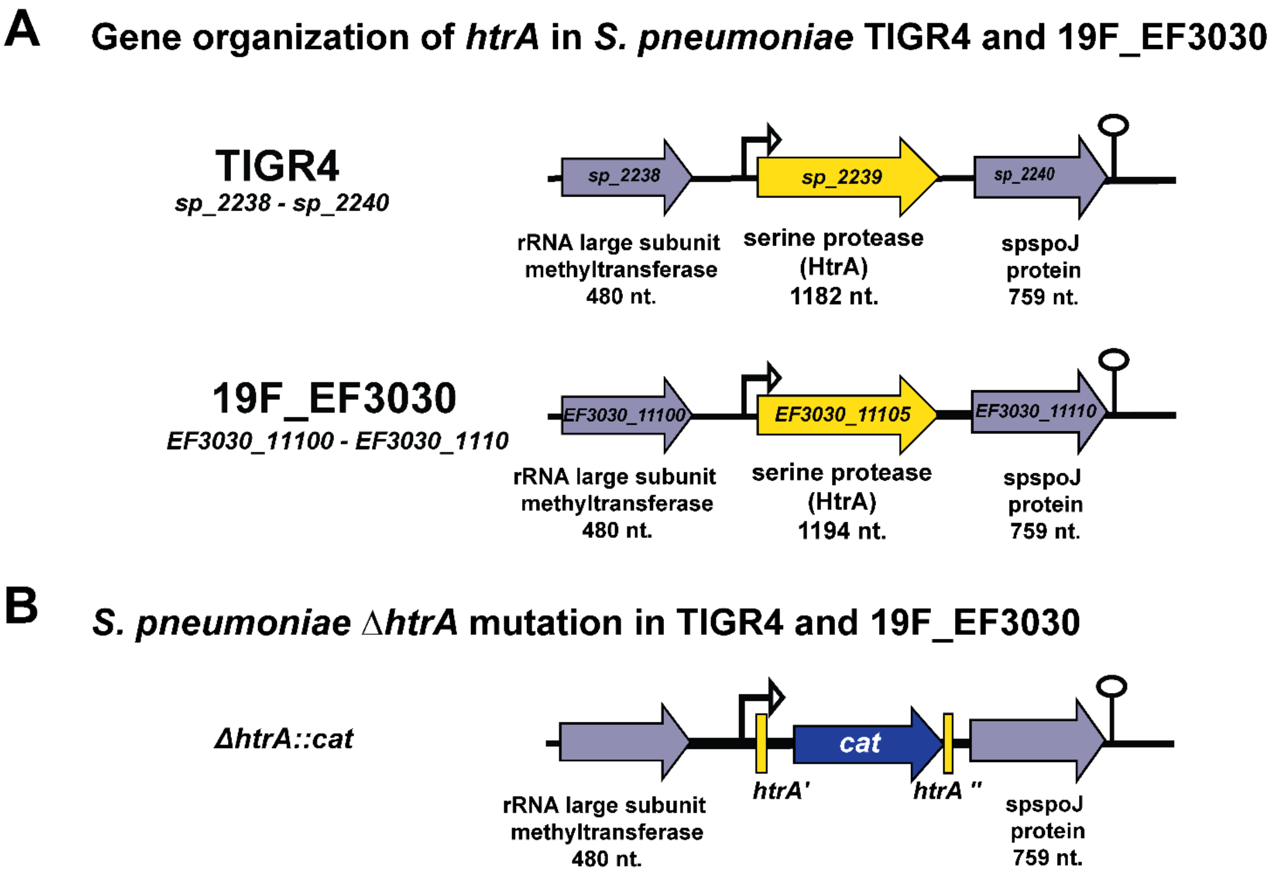

Figure S3. Genetic organization of *htrA* in *S. pneumoniae* TIGR4 and 19F_EF3030.
(A) The chromosomal localization of the *htrA* (high-temperature requirement gene: *sp_2239* in TIGR4 and *EF3030_11105* in 19F). Gene and protein sequences show a 100.0% identity. The gene upstream of *htrA* encodes for an rRNA subunit methyl-transferase. The downstream gene is annotated as ParB/RepB/Spo0J family partition protein. Arrowheads indicate the gene's orientation, the predicted putative promoters (black arrowheads), and the transcription termination signals are shown as loops.
(B) Schematic model of *htrA* mutants constructed by insertion-deletion mutagenesis in TIGR4 and 19F shows the *cat* gene cassette inserted in the *htrA* gene sequence.

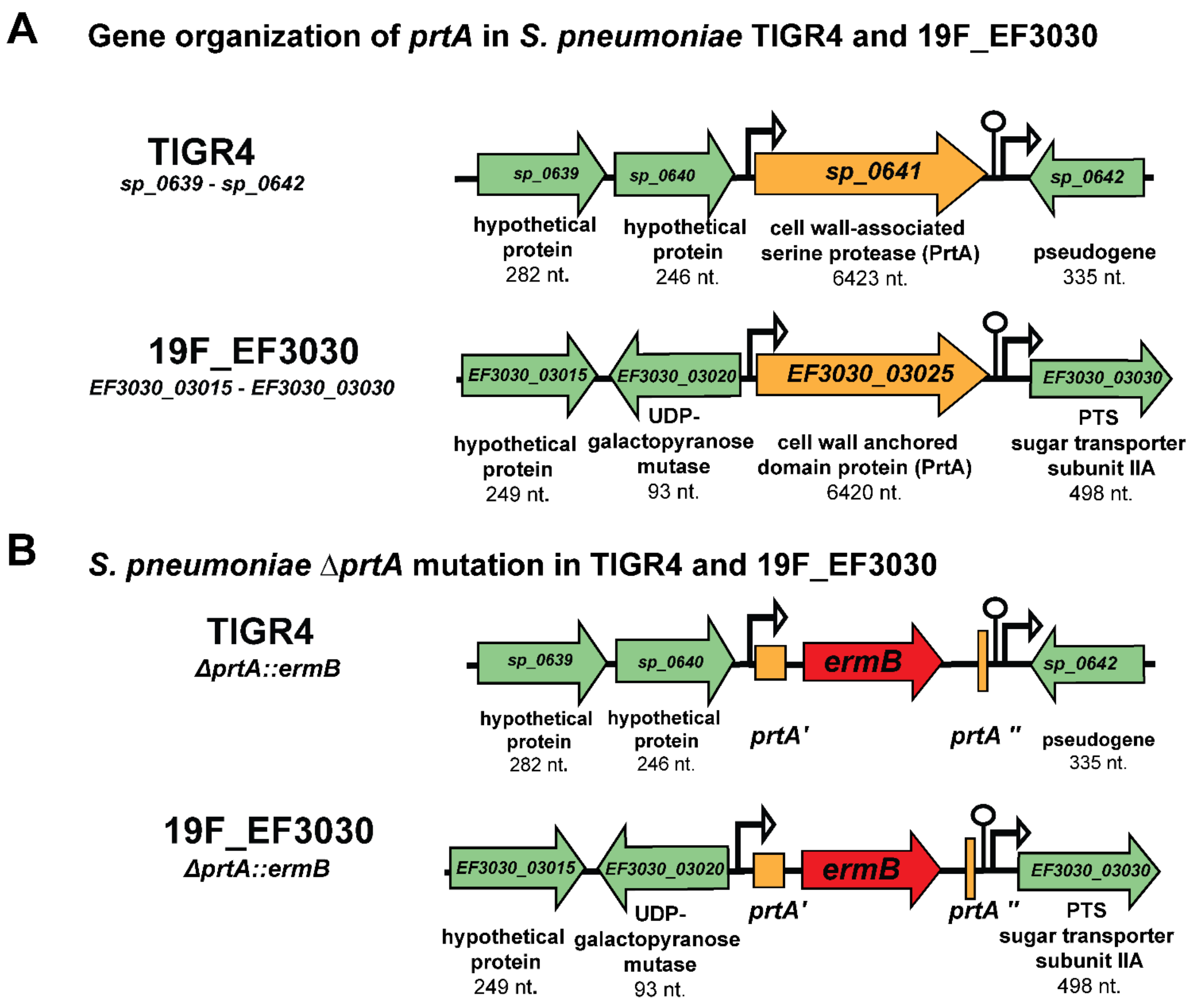

Figure S4. Genetic organization of *prtA* in *S. pneumoniae* in TIGR4 and 19F_EF3030.
(A) The chromosomal localization of the *prtA* gene encoding for the cell wall-associated serine protease (*sp_0641*, *EF3030_03025*). Gene and protein sequences are 100.0% identical. In TIGR4, the gene downstream of *prtA* is a pseudogene, while in 19F_*EF3030_03030*, the gene encodes for a PTS sugar transporter subunit. The genes upstream of *prtA* (*sp_0640*, 246 nt., and *sp_0639*, 282 nt) in TIGR4 encode for hypothetical proteins. 19F_EF3030 shows different upstream genes compared to TIGR4; these genes are *EF3030_03015* and *EF3030_03020*, which encode for a hypothetical protein or UDP-galactopyranose mutase. Arrowheads indicate the gene's orientation, the predicted putative promoters (black arrowheads), and the transcription termination signals are shown as loops.
(B) Schematic model of *prtA* mutants constructed by insertion-deletion mutagenesis in TIGR4 and 19F shows the *ermB* gene cassette inserted in the *prtA* gene sequence.

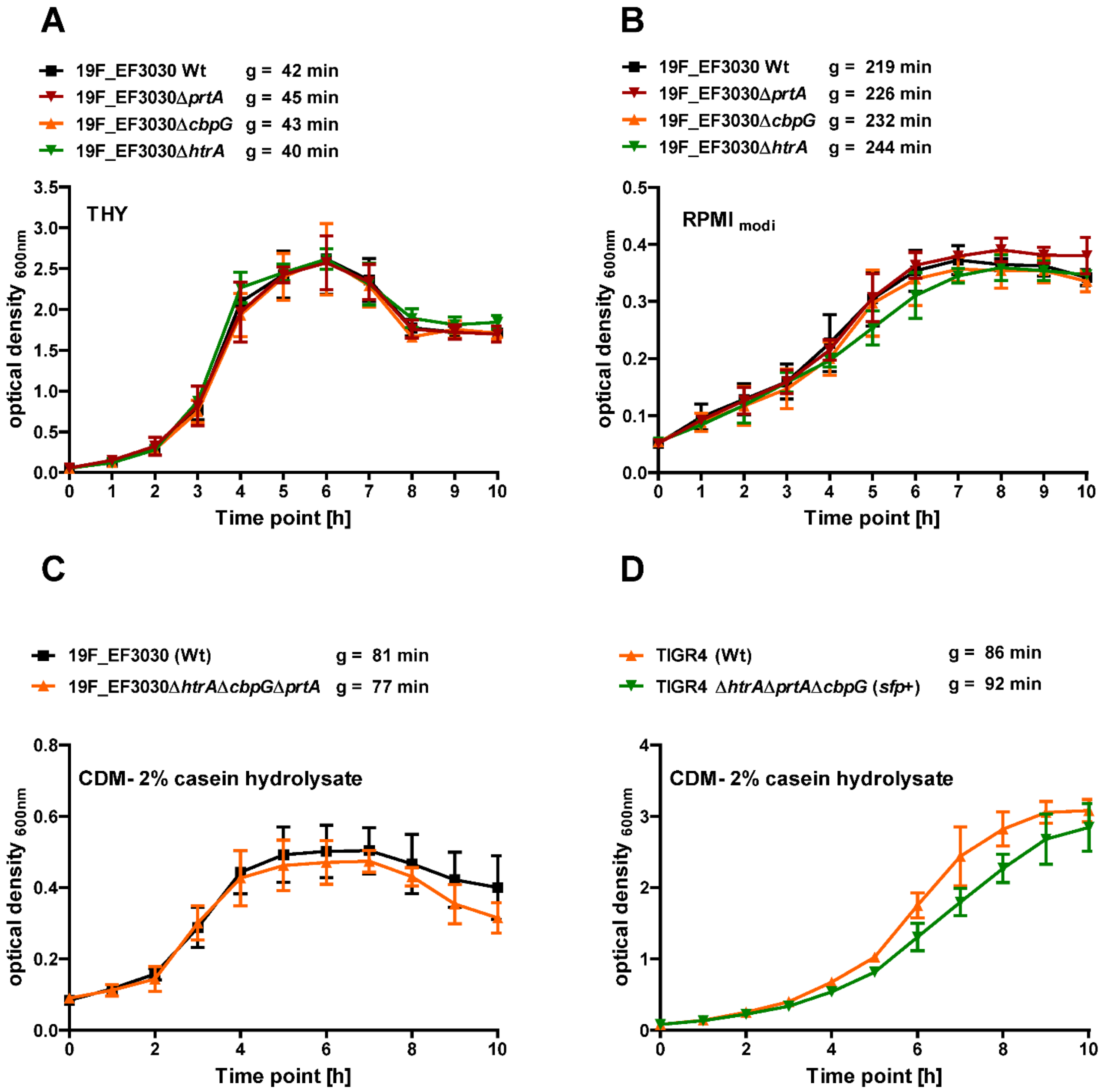

Figure S5. Growth of pneumococcal serine protease mutants in different culture media

(A, B) *S. pneumoniae* 19F_EF3030 wild-type and single isogenic mutants were cultured at 37°C in THY and chemically defined medium ($RPMI_{modi}$).

(C, D) 19F_EF3030 or its isogenic mutant lacking all serine proteases as well as TIGR4 wild-type and its SFP positive triple mutant were grown in CDM without the essential amino acid solution I and II, supplemented instead with 2% casein hydrolysate. Error bars represent SD (n = 4). The symbol "g" indicates the generation time, calculated from four biological replicates. The data were statistically analyzed using a two-way ANOVA analysis. **L-amino acid I:** alanine, asparagine acid, asparagine, glutamine acid, glycine, isoleucine, leucine, lysine, proline, hydroxyproline, serine, valine, glutamine, and threonine. **L-amino acid II:** arginine, histidine, methionine, phenylalanine, tryptophan, and tyrosine.

Table S1. Growth rates of *Streptococcus pneumoniae* wild-type and isogenic mutants in THY and $RPMI_{modi}$ medium.

Pneumococcal strain	Growth rate (μ) in THY [min^{-1}]	Growth rate (μ) in $RPMI_{modi}$ [min^{-1}]
**19F_EF3030 (wild type)**	0.016	0.0031
19F_EF3030Δ*prtA*	0.015	0.0030
19F_EF3030Δ*htrA*	0.017	0.0028
19F_EF3030Δ*cbpG*	0.016	0.0028
**19F_EF3030 (wild type)**	0.0146	0.0045
19FΔ*htrA*Δ*cbpG* (*prtA*+)	0.0149	0.0045
19FΔ*prtA*Δ*cbpG* (*htrA*+)	0.0176	0.0048
19FΔ*prtA*Δ*htrA* (*cbpG*+)	0.0135	0.0041
19FΔ*htrA*Δ*cbpG*Δ*prtA* (*All proteases*)	0.0142	0.0052
**TIGR4Δ*cps* (wild type)**	0.0099	0.018
TIGR4Δ*cps*Δ*htrA*Δ*cbpG*Δ*sfp* (*prtA*+)	0.0096	0.013
TIGR4Δ*cps*Δ*prtA*Δ*cbpG*Δ*sfp* (*htrA*+)	0.0091	0.011
TIGR4Δ*cps*Δ*prtA*Δ*htrA*Δ*sfp* (*cbpG*+)	0.0090	0.009
TIGR4Δ*cps*Δ*htr*AΔ*prt*AΔ*cbp*G (*sfp*+)	0.0085	0.010
**TIGR4*lux* (wild type)**	0.0119	0.0041
TIGR4*lux*Δ*htrA*Δ*cbpG*Δ*sfp* (*prtA*+)	0.0123	0.0041
TIGR4*lux*Δ*prtA*Δ*cbpG*Δ*sfp* (*htrA*+)	0.0113	0.0041
TIGR4*lux*Δ*htrA*Δ*prtA*Δ*sfp* (*cbpG*+)	0.0081	0.0045
TIGR4*lux*Δ*htr*AΔ*prt*AΔ*cbp*G (*sfp*+)	0.0083	0.0045

Table S2. Colony-forming units (CFU) of adherent pneumococci counted on blood agar plates after 4 h of infection of Detroit-562 cells

Pneumococcal strain	Adherent bacteria CFU\ml per 2.5 x 10^5 cells	
	Mean ± SD	*P*-value
**19F_EF3030 (wild type)**	1162 ± 928.4	-
19FΔ*htrA*Δ*cbpG* (*prtA*+)	144.2 ± 140.1	0,0162
19FΔ*prtA*Δ*cbpG* (*htrA*+)	154.8 ± 151.4	0,0162
19FΔ*prtA*Δ*htrA* (*cbpG*+)	147.1 ± 175.3	0,0162
19FΔ*htrA*Δ*cbpG*Δ*prtA* (*non-function serine proteases*)	98.71 ± 109.6	0,0040
**TIGR4Δ*cps* (wild type)**	4870 ± 1294	-
TIGR4Δ*cps*Δ*htrA*Δ*cbpG*Δ*sfp* (*prtA*+)	2365 ± 1428	0.0591
TIGR4Δ*cps*Δ*prtA*Δ*cbpG*Δ*sfp* (*htrA*+)	1491 ± 1253	0.0294
TIGR4Δ*cps*Δ*prtA*Δ*htrA*Δ*sfp* (*cbpG*+)	1424 ± 8630	0.0294
TIGR4Δ*cps*Δ*htr*AΔ*prt*AΔ*cbp*G (*sfp*+)	8307 ± 2650	0.0294

*P value less than 0.05 was taken as statistically significant.

Table S3. Colony-forming units (CFU) from nasopharyngeal lavage counted on blood agar plates

Pneumococcal strains	CFU\ml in the nasopharyngeal lavage							
	**Median Day 2**	***P*-value**	**Median Day3**	***P*-value**	**Median Day 7**	***P*-value**	**Median Day 14**	***P*-value**
**19F_EF3030 (wild type)**	291000		210000		147000		23300	
19FΔ*htrA*Δ*cbpG* (*prtA*+)	31700	0,0004	27600	0,0004	25800	0,0005	7070	0,0163
19FΔ*prtA*Δ*cbpG* (*htrA*+)	53700	0,0025	69200	0,0153	25200	0,0009	9950	0,0630
19FΔ*prtA*Δ*htrA* (*cbpG*+)	21600	0,0005	16700	0,0003	20000	0,0009	6060	0,0066
19FΔ*htrA*Δ*cbpG*Δ*prtA*	33800	0,0005	35400	0,0012	36000	0,0081	1860	0,0015

**P* value less than 0.05 was taken as statistically significant.

Table S4. Colony-forming units from bronchoalveolar lavage counted on blood agar plates

Pneumococcal strain	CFU\ml in the nasopharyngeal wash							
	**Median Day 2**	***P*-value**	**Median Day3**	***P*-value**	**Median Day 7**	***P*-value**	**Median Day 14**	***P*-value**
**19F_EF3030 (wild type)**	564,0		1795		6408		33,00	
19FΔ*htrA*Δ*cbpG* (*prtA*+)	15,00	0,1443	215,8	0,5753	30,00	0,0040	1,000	0,7819
19FΔ*prtA*Δ*cbpG* (*htrA*+)	175,0	0,8519	50,00	0,0224	16,65	0,0852	233,0	0,5841
19FΔ*prtA*Δ*htrA* (*cbpG*+)	207,0	0,4327	108,0	0,1916	133,0	0,0733	1,000	0,0370
19FΔ*htrA*Δ*cbpG*Δ*prtA*	1240	0,8813	425,0	0,6815	1,000	0,0911	1,000	0,0156

**P* value less than 0.05 was taken as statistically significant.

1 Pneumococcal serine protease amino acid sequences

1.1 HtrA amino acid sequences of *S. pneumoniae sp_2239* in *TIGR4* and *EF3030_11105* in 19F proteins are identical (protein accession no. AAK76286.1 and QBF69928.1). The signal peptide sequence (31 aa) is marked in blue, the serine protease catalytic domain (182 aa) position 96-277 is marked in red, PDZ domain (87 aa) position 289-375 is marked in green.

MKHLKTFYKKWFQLLVVIVISFFSGALGSFSITQLTQKSSVNNSNNNSTITQTAYKNENSTTQAVNKVKDAVVSVITYSAN
RQNSVFGNDDTDTDSQRISSEGSGVIYKKNDKEAYIVTNNHVINGASKVDIRLSDGTKVPGEIVGADTFSDIAVVKISSEK
VTTVAEFGDSSKLTVGETAIAIGSPLGSEYANTVTQGIVSSLNRNVSLKSEDGQAISTKAIQTDTAINPGNSGGPLINIQG
QVIGITSSKIATNGGTSVEGLGFAIPANDAINIIEQLEKNGKVTRPALGIQMVNLSNVSTSDIRRLNIPSNVTSGVIVRSV
QSNMPANGHLEKYDVITKVDDKEIASSTDLQSALYNHSIGDTIKITYYRNGKEETTSIKLNKSSGDLES

1.2 PrtA amino acid sequences of *S. pneumoniae sp_0641* in TIG4 and *EF3030_03025* in 19F proteins are identical (protein accession no. AAK74791.1 and QBF68585.1). The signal peptide sequence (27 aa) is marked in blue, the serine protease catalytic domain (542 aa) position 223-764 is marked in red, DUF 1034 domain (140 aa) position 795-934 is marked in green. The C-terminal LPKTG anchoring motif (42) is marked in yellow.

MKKSTVLSLTTAAVILAAYAPNEVVLADTSSSEDALNISDKEKVAENKEKHENIHSAMETSQDFKEKKTAVIKEKEVVSK
NPVIDNNTSNEEAKIKEENSNKSQGDYTDSFVNKNTENPKKEDKVVYIAEFKDKESGEKAIKELSSLKNTKVLYTYDRIF
NGSAIETTPDNLDKIKQIEGISSVERAQKVQPMMNHARKEIGVEEAIDYLKSINAPFGKNFDGRGMVISNIDTGTDYRHK
AMRIDDDAKASMRFKKEDLKGTDKNYWLSDKIPHAFNYYNGGKITVEKYDDGRDYFDPHGMHIAGILAGNDTEQDIKNFN
GIDGIAPNAQIFSYKMYSDAGSGFAGDETMFHAIEDSIKHNVDVVSVSSGFTGTGLVGEKYWQAIRALRKAGIPMVVATG
NYATSASSSSWDLVANNHLKMTDTGNVTRTAAHEDAIAVASAKNQTVEFDKVNIGGESFKYRNIGAFFDKSKITTNEDGT
KAPSKLKFVYIGKGQDQDLIGLDLRGKIAVMDRIYTKDLKNAFKKAMDKGARAIMVVNTVNYYNRDNWTELPAMGYEADE
GTKSQVFSISGDDGVKLWNMINPDKKTEVKRNNKEDFKDKLEQYYPIDMESFNSNKPNVGDEKEIDFKFAPDTDKELYKE
DIIVPAGSTSWGPRIDLLLKPDVSAPGKNIKSTLNVINGKSTYGYMSGTSMATPIVAASTVLIRPKLKEMLERPVLKNLK
GDDKIDLTSLTKIALQNTARPMMDATSWKEKSQYFASPRQQGAGLINVANALRNEVVATFKNTDSKGLVNSYGSISLKEI
KGDKKYFTIKLHNTSNRPLTFKVSASAITTDSLTDRLKLDETYKDEKSPDGKQIVPEIHPEKVKGANITFEHDTFTIGAN
SSFDLNAVINVGEAKNKNKFVESFIHFESVEEMEALNSNGKKINFQPSLSMPLMGFAGNWNHEPILDKWAWEEGSRSKTL
GGYDDDGKPKIPGTLNKGIGGEHGIDKFNPAGVIQNRKDKNTTSLDQNPELFAFNNEGINAPSSSGSKIANIYPLDSNGN
PQDAQLERGLTPSPLVLRSAEEGLISIVNTNKEGENQRDLKVISREHFIRGILNSKSNDAKGIKSSKLKVWGDLKWDGLI
YNPRGREENAPESKDNQDPATKIRGQFEPIAEGQYFYKFKYRLTKDYPWQVSYIPVKIDNTAPKIVSVDFSNPEKIKLIT
KDTYHKVKDQYKNETLFARDQKEHPEKFDEIANEVWYAGAALVNEDGEVEKNLEVTYAGEGQGRNRKLDKDGNTIYEIKG
AGDLRGKIIEVIALDGSSNFTKIHRIKFANQADEKGMISYYLVDPDQDSSKYQKLGEIAESKFKNLGNGKEGSLKKDTTG
VEHHHQENEESIKEKSSFTIDRNISTIRDFENKDLKKLIKKKFREVDDFTSETGKRMEEYDYKYDDKGNIIAYDDGTDLE
YETEKLDEIKSKIYGVLSPSKDGHFEILGKISNVSKNAKVYYGNNYKSIEIKATKYDFHSKTMTFDLYANINDIVDGLAF
AGDMRLFVKDNDQKKAEIKIRMPEKIKETKSEYPYVSSYGNVIELGEGDLSKNKPDNLTKMESGKIYSDSEKQQYLLKDN
IILRKGYALKVTTYNPGKTDMLEGNGVYSKEDIAKIQKANPNLRALSETTIYADSRNVEDGRSTQSVLMSALDGFNIIRY
QVFTFKMNDKGEAIDKDGNLVTDSSKLVLFGKDDKEYTGEDKFNVEAIKEDGSMLFIDTKPVNLSMDKNYFNPSKSNKIY
VRNPEFYLRGKISDKGGFNWELRVNESVVDNYLIYGDLHIDNTRDFNIKLNVKDGDIMDWGMKDYKANGFPDKVTDMDGN
VYLQTGYSDLNAKAVGVHYQFLYDNVKPEVNIDPKGNTSIEYADGKSVVFNINDKRNNGFDGEIQEQHIYINGKEYTSFN
DIKQIIDKTLNIKIVVKDFARNTTVKEFILNKDTGEVSELKPHRVTVTIQNGKEMSSTIVSEEDFILPVYKGELEKGYQF
DGWEISGFEGKKDAGYVINLSKDTFIKPVFKKIEEKKEEENKPTFDVSKKKDNPQVNHSQLNESHRKEDLQREEHSQKSD
STKDVTATVLDKNNISSK**STTNNPNKLPKTGTASGAQTLLAAGIMFIVGIFLGLKKKNQD**

1.3 SFP amino acid sequences of *S. pneumoniae sp_1954* in TIGR4 (protein accession number ABC75782.1). The signal peptide sequence (22 aa) is marked in blue, the serine protease catalytic domain (295 aa) position 167-461 is marked in red

MKKKYWTLAILFFCLFNNSVTAQEIPKNLDGNITHTQTSESFSESDEKQVDYSNKNQEEVDQNKFRIQIDKTELFVTTDKH
LEKNCCKLELEPQINNDIVNSESNNLLGEDNLDNKIKENVSHLDNRGGNIEHDKDNLESSIVRKYEWDIDKVTGGGESYKL
YSKS**NSKVSIAILDSGVDLQNTGLLKNLSNHSKNYVPNKGYLGKEEGEEGIISDIQDRLGHGTAVVAQIVGDDNINGVNPH**
VNINVYRIFGKSSASPDWIVKAIFDAVDDGNDIINLSTGQYLMIDGEYEDGTNDFETFLKYKKAIDYANQKGVIIVAALGN
DSLNVSNQSDLLKLISSRKKVRKPGLVVDVPSYFSSTISVGGIDRLGNLSDFSNKGDSDAIYAPAGSTLSLSELGLNNFIN
AEKYKEDWIFSATLGGYTYLYGNSFAAPKVSGAIAMIIDKYKLKDQPYNYMFVKKFWKKHYQ

1.4 CbpG amino acid sequences of *S. pneumoniae sp_0390 in* TIGR4 and *EF3030_01920* in 19F protein are highly homologous in TIGR4 and 19F (protein accession no. AAK74556.1 and QBF69943.1). The trypsin-like serine protease catalytic domain (184 aa) position 14-197 is marked in red, the repeats of the CBM (CW1 position 207-221), (CW2 position 226-245), and (CW3 position 246-265) are marked in orange. At the C-terminal region (in light blue, position 267-285), it is probably involved in binding to choline resides of teichoic acids.

MVLSKYYGVADGMNVEGRGSANFIKDNVLITAAHNYYRHDYGKEADDIYVLPAVSPSQEPFGKIKVKEVRYLKEFRNLNSK
DAREYDLALLILEEPIGAKLGTLGLPTSQKNLTGITVTITGYPSYNFKIHQMYTDKKQVLSDDGMFLDYQVDTLEGSSGST
VYDASHRVVGVHTLGDGANQINSAVKLNERNLPFIYSVLKGYSL**EGWKKINGSWYHYRQ**HDKQ**TGWQEINDTWYYLDSSGK**
MLTDWQKVNGKWYYLNSNGAMVTGSQTIDGKV**Y**NFASSGE**WI**

TIGR4 and 19F_EF3030 HtrA protein sequence alignment

```
sp_2239           ----MKHLKTFYKKWFQLLVVIVISFFSGALGSFSITQLTQKSSVNNSNNNSTITQTAYK      56
EF3030_11105      MEANMKHLKTFYKKWFQLLVVIVISFFSGALGSFSITQLTQKSSVNNSNNNSTITQTAYK      60
                      ********************************************************

sp_2239           NENSTTQAVNKVKDAVVSVITYSANRQNSVFGNDDTDTDSQRISSEGSGVIYKKNDKEAY      116
EF3030_11105      NENSTTQAVNKVKDAVVSVITYSANRQNSVFGNDDTDTDSQRISSEGSGVIYKKNDKEAY      120
                  ************************************************************

sp_2239           IVTNNHVINGASKVDIRLSDGTKVPGEIVGADTFSDIAVVKISSEKVTTVAEFGDSSKLT      176
EF3030_11105      IVTNNHVINGASKVDIRLSDGTKVPGEIVGADTFSDIAVVKISSEKVTTVAEFGDSSKLT      180
                  ************************************************************

sp_2239           VGETAIAIGSPLGSEYANTVTQGIVSSLNRNVSLKSEDGQAISTKAIQTDTAINPGNSGG      236
EF3030_11105      VGETAIAIGSPLGSEYANTVTQGIVSSLNRNVSLKSEDGQAISTKAIQTDTAINPGNSGG      240
                  ************************************************************

sp_2239           PLINIQGQVIGITSSKIATNGGTSVEGLGFAIPANDAINIIEQLEKNGKVTRPALGIQMV      296
EF3030_11105      PLINIQGQVIGITSSKIATNGGTSVEGLGFAIPANDAINIIEQLEKNGKVTRPALGIQMV      300
                  ************************************************************

sp_2239           NLSNVSTSDIRRLNIPSNVTSGVIVRSVQSNMPANGHLEKYDVITKVDDKEIASSTDLQS      356
EF3030_11105      NLSNVSTSDIRRLNIPSNVTSGVVVRSVQSNMPANGHLEKYDVITKVDDKEIASSTDLQS      360
                  ***********************:************************************

sp_2239           ALYNHSIGDTIKITYYRNGKEETTSIKLNKSSGDLES    393
EF3030_11105      ALYNHSIGDTIKITYYRNGKEETTSIKLNKSSGDLES    397
                  *************************************
```

TIGR4 and 19F_EF3030 PrtA protein sequence alignment

```
sp_0641TIG4       MKKSTVLSLTTAAVILAAYAPNEVVLADTSSSEDALNISDKEKVAENKEKHENIHSAMET      60
EF3030_03025      MKKSTVLSLTTAAVILAAYAPNEVVLADTSSSEDALSISDKEKVAENKEKHKDIHNAIET      60
                  ************************************.**************::**.*:**

sp_0641TIG4       SQDFKEKKTAVIKEKEVVSKNPVIDNNTSNEEAKIKEENSNKSQGDYTDSFVNKNTENPK      120
EF3030_03025      SKDTEEKKTTIIEEKEVVSKNPVIDTKTSNEEAKIKEENSNKSQGDHTDSFVNKNTENPK      120
                  *:* :****::*:************.:*******************:*************

sp_0641TIG4       KEDKVVYIAEFKDKESGEKAIKELSSLKNTKVLYTYDRIFNGSAIETTPDNLDKIKQIEG      180
EF3030_03025      KEDKVVYIAEFKDKESGSKAIKELSSLKNTKVLYTYDRIFNGGAIETTQDNLNKIKQIEG      180
                  *****************.************************.***** ***:*******

sp_0641TIG4       ISSVERAQKVQPMMNHARKEIGVEEAIDYLKSINAPFGKNFDGRGMVISNIDTGTDYRHK      240
EF3030_03025      ITSVERAQKVQPMMNHARKEIGVEEAIDYLKSINAPFGKNFDGRGMVISNIDTGTDYRHK      240
                  *:**********************************************************

sp_0641TIG4       AMRIDDDAKASMRFKKEDLKGTDKNYWLSDKIPHAFNYYNGGKITVEKYDDGRDYFDPHG      300
EF3030_03025      AMRIDDDAKASMRFKKEDLKGTDKNYWLSDKIPHAFNYYNGGKITVEKYDDGRDYFDPHG      300
                  ************************************************************

sp_0641TIG4       MHIAGILAGNDTEQDIKNFNGIDGIAPNAQIFSYKMYSDAGSGFAGDETMFHAIEDSIKH      360
EF3030_03025      MHIAGILAGNDTEQDIKNFNGIDGIAPNAQIFSYKMYSDAGSGFAGDETMFHAIEDSIKH      360
                  ************************************************************

sp_0641TIG4       NVDVVSVSSGFTGTGLVGEKYWQAIRALRKAGIPMVVATGNYATSASSSSWDLVANNHLK      420
EF3030_03025      NVDVVSVSSGFTGTGLVGEKYWQAIRALRKAGIPMVVATGNYATSASSSSWDLVANNHLK      420
                  ************************************************************

sp_0641TIG4       MTDTGNVTRTAAHEDAIAVASAKNQTVEFDKVNIGGESFKYRNIGAFFDKSKITTNEDGT      480
EF3030_03025      MTDTGNVTRTAAHEDAIAVASAKNQTVEFDKVNIGGESFKYRNIGAFFDKNKITTNEDGT      480
                  **************************************************.*********

sp_0641TIG4       KAPSKLKFVYIGKGQDQDLIGLDLRGKIAVMDRIYTKDLKNAFKKAMDKGARAIMVVNTV      540
EF3030_03025      KAPSKLKFVYIGKGQDQDLIGLDLRGKIAVMDRIYTKDLKNAFKKAMDKGARAIMVVNTV      540
                  ************************************************************

sp_0641TIG4       NYYNRDNWTELPAMGYEADEGTKSQVFSISGDDGVKLWNMINPDKKTEVKRNNKEDFKDK      600
EF3030_03025      NYYNRDNWTELPAMGYEADEGTKSQVFSISGDDGVKLWNMINPNKKTEVKRNNKEDFKDK      600
                  *******************************************:****************

sp_0641TIG4       LEQYYPIDMESFNSNKPNVGDEKEIDFKFAPDTDKELYKEDIIVPAGSTSWGPRIDLLLK      660
EF3030_03025      LEQYYPIDMESFNSNKPNVGDEKEIDFKFAPDTDKELYKEDIIVPAGSTSWGPRIDLLLK      660
                  ************************************************************

sp_0641TIG4       PDVSAPGKNIKSTLNVINGKSTYGYMSGTSMATPIVAASTVLIRPKLKEMLERPVLKNLK      720
EF3030_03025      PDVSAPGKNIKSTLNVINGKSTYGYMSGTSMATPIVAASTVLIRPKLKEMLERPVLKNLK      720
                  ************************************************************

sp_0641TIG4       GDDKIDLTSLTKIALQNTARPMMDATSWKEKSQYFASPRQQGAGLINVANALRNEVVATF      780
EF3030_03025      GDDKIDLTSLTKIALQNTARPMMDATSWKEKSQYFASPRQQGAGLINVANALRNEVVATF      780
                  ************************************************************

sp_0641TIG4       KNTDSKGLVNSYGSISLKEIKGDKKYFTIKLHNTSNRPLTFKVSASAITTDSLTDRLKLD      840
EF3030_03025      KNTDSKGLVNSYGSISLKEIKGDKKYFTIKLHNTSNRPLTFKVSASAITTDSLTDRLKLD      840
                  ************************************************************

sp_0641TIG4       ETYKDEKSPDGKQIVPEIHPEKVKGANITFEHDTFTIGANSSFDLNAVINVGEAKNKNKF      900
EF3030_03025      ETYKDEKSPDGKQIVPEIHPEKVKGANITFEHDTFTIGANSSFDLNAVINVGEAKNKNKF      900
                  ************************************************************
```

```
sp_0641TIG4      VESFIHFESVEEMEALNSNGKKINFQPSLSMPLMGFAGNWNHEPILDKWAWEEGSRSKTL    960
EF3030_03025     VESFIHFESVEEMEALNSNGKKINFQPSLSMPLMGFAGNWNHEPILDKWAWEEGSRSKTL    960
                 ************************************************************

sp_0641TIG4      GGYDDDGKPKIPGTLNKGIGGEHGIDKFNPAGVIQNRKDKNTTSLDQNPELFAFNNEGIN    1020
EF3030_03025     GGYDDDGKPKIPGTLNKGIGGEHGIDKFNPAGVIQNRKDKNTTSLDQNPELFAFNNQGIN    1020
                 ********************************************************:***

sp_0641TIG4      APSSSGSKIANIYPLDSNGNPQDAQLERGLTPSPLVLRSAEEGLISIVNTNKEGENQRDL    1080
EF3030_03025     APSSSGSKIANIYPLDSNGNPQDAQLERGLTPSPLVLRSAEEGLISIVNTNKEGENQRDL    1080
                 ************************************************************

sp_0641TIG4      KVISREHFIRGILNSKSNDAKGIKSSKLKVWGDLKWDGLIYNPRGREENAPESKDNQDPA    1140
EF3030_03025     KVISREHFIRGILNSKSNDAKGIKSSKLKVWGDLKWDGLIYNPRGREENAPESKDNQDPA    1140
                 ************************************************************

sp_0641TIG4      TKIRGQFEPIAEGQYFYKFKYRLTKDYPWQVSYIPVKIDNTAPKIVSVDFSNPEKIKLIT    1200
EF3030_03025     TKIRGQFEPIAEGQYFYKFKYRLTKDYPWQVSYIPVKIDNTAPKIVSVDFSNPEKIKLIT    1200
                 ************************************************************

sp_0641TIG4      KDTYHKVKDQYKNETLFARDQKEHPEKFDEIANEVWYAGAALVNEDGEVEKNLEVTYAGE    1260
EF3030_03025     KDTYHKVKDQYKNETLFARDQKEHPEKFDEIANEVWYAGAALVNEDGEVEKNLEVTYAGE    1260
                 ************************************************************

sp_0641TIG4      GQGRNRKLDKDGNTIYEIKGAGDLRGKIIEVIALDGSSNFTKIHRIKFANQADEKGMISY    1320
EF3030_03025     GQGRNRKLDKDGNTIYEIKGAGDLRGKIIEVIALDGSSNFTKIHRIKFANQADEKGMISY    1320
                 ************************************************************

sp_0641TIG4      YLVDPDQDSSKYQKLGEIAESKFKNLGNGKEGSLKKDTTGVEHHHQENEESIKEKSSFTI    1380
EF3030_03025     YLVDPDQDSSKYQKLGEIAESKFKNLGNGKEGSLKKDTTGVEHHHQENEESIKEKSSFTI    1380
                 ************************************************************

sp_0641TIG4      DRNISTIRDFENKDLKKLIKKKFREVDDFTSETGKRMEEYDYKYDDKGNIIAYDDGTDLE    1440
EF3030_03025     DRNISTIRDFENKDLKKLIKKKYKEEDDFVT-GGKRTVELDYKYDDKGNITAYEDESALE    1439
                 **********************::* ***.:  ***  * ********** **:* : **

sp_0641TIG4      YETEKLDEIKSKIYGVLSPSKDGHFEILGKISNVSKNAKVYYGNNYKSIEIKATKYDFHS    1500
EF3030_03025     YETEKFDEIKSKIYGVLSPSKDGHFEILGKISNVSKNAKVYYGNNYKFIEIKATKYDSHS    1499
                 *****:***************************************** ********* **

sp_0641TIG4      KTMTFDLYANINDIVDGLAFAGDMRLFVKDNDQKKAEIKIRMPEKIKETKSEYPYVSSYG    1560
EF3030_03025     KTMTFDLYANINDIVDGLAFAGDMRFFVKDDDRIKAETKIRMPEKNKETKAEYPYVSSYG    1559
                 *************************:****:*:* *** ******* ****:*********

sp_0641TIG4      NVIELGEGDLSKNKPDNLTKMESGKIYSDSEKQQYLLKDNIILRKGYALKVTTYNPGKTD    1620
EF3030_03025     NVIELGEGDLSKNKPDNLTKMESGKIYSDSEKQQYLLKDNIILRKGYALKVTTYNPGKTD    1619
                 ************************************************************

sp_0641TIG4      MLEGNGVYSKEDIAKIQKANPNLRALSETTIYADSRNVEDGRSTQSVLMSALDGFNIIRY    1680
EF3030_03025     MLEGNGVYSKEDIAKIQKANPNLRVLSETTIYADSRNVEDGRSTQAVLMSALDGFNIIRY    1679
                 ************************.********************:**************

sp_0641TIG4      QVFTFKMNDKGEAIDKDGNLVTDSSKLVLFGKDDKEYTGEDKFNVEAIKEDGSMLFIDTK    1740
EF3030_03025     QVFTFKMNDKGEAIDKDGNLVTDSSKLVLFGKDDKEYTGEDKSNVEAIKEDGSMLFIDTK    1739
                 ****************************************** *****************

sp_0641TIG4      PVNLSMDKNYFNPSKSNKIYVRNPEFYLRGKISDKGGFNWELRVNESVVDNYLIYGDLHI    1800
EF3030_03025     PVNLSMDKNYFNPSKSNKIYVRNPEFYLRGKISDKGGFNWELRVNESVVDNYLIYGDLHI    1799
                 ************************************************************

sp_0641TIG4      DNTRDFNIKLNVKDGDIMDWGMKDYKANGFPDKVTDMDGNVYLQTGYSDLNAKAVGVHYQ    1860
EF3030_03025     DNTRDFNIKLNVKDGDIMDWGMKDYKANGFPDKVTDMDGNVYLQTGYSDLNAKAVGVHYQ    1859
                 ************************************************************

sp_0641TIG4      FLYDNVKPEVNIDPKGNTSIEYADGKSVVFNINDKRNNGFDGEIQEQHIYINGKEYTSFN    1920
```

```
EF3030_03025      FLYDNVKPEVNIDPKGNTSIEYADGKSVVFNINDKRNNGFDGEIQEQHIYVNGKEYTSFD      1919
                  **************************************************:*********:

sp_0641TIG4       DIKQIIDKTLNIKIVVKDFARNTTVKEFILNKDTGEVSELKPHRVTVTIQNGKEMSSTIV      1980
EF3030_03025      DIKQITDKTLNIKIVVKDFARNTTVKEFILNKDTGEVSELKPHRVTVTIQNGKEMSSTIV      1979
                  ***** ******************************************************

sp_0641TIG4       SEEDFILPVYKGELEKGYQFDGWEISGFEGKKDAGYVINLSKDTFIKPVFKKIEEKKEEE      2040
EF3030_03025      SEEDFILPVYKGELEKGYQFDGWEISGFEGKKDAGYVINLSKDTFIKPVFKKIEEKKEEE      2039
                  ************************************************************

sp_0641TIG4       NKPTFDVSKKKDNPQVNHSQLNESHRKEDLQREEHSQKSDSTKDVTATVLDKNNISSKST      2100
EF3030_03025      NKPTFDVSKKKDNPQVNHSQLNESHRKEDLQREDHSQKSDSTKDVTATVLDKNNISSKST      2099
                  *********************************:**************************

sp_0641TIG4       TNNPNKLPKTGTASGAQTLLAAGIMFIVGIFLGLKKKNQD 2140
EF3030_03025      TNNPNKLPKTGTASGAQTLLAAGIMFIVGIFLGLKKKNQD 2139
                  ****************************************
```

TIGR4 and 19F_EF3030 CbpG protein sequence alignment

```
sp_0390          MVLSKYYGVADGMNVEGRGSANFIKDNVLITAAHNYYRHDYGKEADDIYVLPAVSPSQEP       60
EF3030_01920     ------------MNVEGRGSANFIKDNVLITAAHNYYRHDYGKEADDIYVLPAVSPSQEL       48
                             ***********************************************

sp_0390          FGKIKVKEVRYLKEFRNLNSKDAREYDLALLILEEPIGAKLGTLGLPTSQKNLTGITVTI       120
EF3030_01920     FGKIKVKEVRYLKEFRNLNSKDAREYDLALLILEEPIGAKLGTLGLPTSQKNLTGITVTI       108
                 ************************************************************

sp_0390          TGYPSYNFKIHQMYTDKKQVLSDDGMFLDYQVDTLEGSSGSTVYDASHRVVGVHTLGDGA       180
EF3030_01920     TGYPSYNFKIHQMYTDKKQVLSDDGMFLDYQVDTLEGSSGSTVYDASHRVVGVHTLGDGA       168
                 ************************************************************

sp_0390          NQINSAVKLNERNLPFIYSVLKGYSLEGWKKINGSWYHYRQHDKQTGWQEINDTWYYLDS       240
EF3030_01920     NQINSAVKLNERNLPFIYSVLKGYSLEGWKKINGSWYHYRQHDKQTGWQEINDTWYYLDS       228
                 ************************************************************

sp_0390          SGKMLTDWQKVNGKWYYLNSNGAMVTGSQTIDGKVYNFASSGEWI     285
EF3030_01920     SGKMLTDWQKVNGKWYYLNSNGAMVTGSQTIDGKVYNFASSGEWI     273
```
